# Rapid monitoring of fermentations: a feasibility study on biological 2,3-butanediol production

**DOI:** 10.1186/s13068-025-02662-1

**Published:** 2025-06-07

**Authors:** Zofia Tillman, Darren J. Peterson, Nancy Dowe, Ed Wolfrum

**Affiliations:** National Renewable Energy Laboratory, 15013 Denver West Pkwy, Golden, CO 80401 USA

**Keywords:** NIRS, Partial least squares, Fermentation, 2,3-butanediol, Process analytical technology

## Abstract

**Background:**

2,3-butanediol (2,3-BDO) is an economically important platform chemical that can be produced by the fermentation of sugars using an engineered strain of Z*ymomonas mobilis*. These fermentations require continuous monitoring and modification of fermentation conditions to maximize 2,3-BDO yields and minimize the production of the undesired coproducts glycerol and acetoin. Because of the time required for sampling and off-line chromatographic measurement of fermentation samples, the ability of fermentation scientists to modify fermentation conditions in a timely manner is limited. The goal of this study was to test if near-infrared spectroscopy (NIRS) along with multivariate statistics could reduce the time needed for this analysis and enable real-time monitoring and control of the fermentation.

**Results:**

In this work we developed partial least squares (PLS) calibration models to predict the concentrations of glucose, xylose, 2,3-BDO, acetoin, and glycerol in fermentations via NIRS using two different spectrometers and two different spectroscopy modalities. We first evaluated the feasibility of rapid NIRS monitoring through experiments where we measured the signals from each analyte of interest and built NIRS-based PLS models using spectra from synthetic samples containing uncorrelated concentrations of these analytes. All analytes showed unique spectral signatures, and this initial modeling showed that all analytes could be detected simultaneously. We then began work with samples from laboratory fermentation experiments and tested the feasibility of regression model development across two spectral collection modalities (at-line and on-line) and two instruments: a laboratory-grade instrument and a low-cost instrument with a more limited spectral range. All modalities showed promise in the ability to monitor *Z. mobilis* fermentations of glucose and xylose to 2,3-BDO. The low-cost instrument displayed a lower signal-to-noise ratio than the laboratory-grade instrument, which led to comparatively lower performance overall, but still provided sufficient accuracy to monitor fermentation trends. While the ease of use of on-line monitoring systems was favored as compared to at-line systems due to the lack of sampling required and potential for automated process control, we observed some decrease in performance due to the additional complexity of the sample matrix.

**Conclusion:**

We have demonstrated that NIRS combined with multivariate analysis can be used for at-line and on-line monitoring of the concentrations of glucose, xylose, 2,3-BDO, acetoin, and glycerol during *Z. mobilis* fermentations. The decrease in signal-to-noise ratio when using a low-cost spectrometer led to greater prediction error than the laboratory-grade spectrometer for at-line monitoring. The on-line monitoring modality showed great promise for real time process control via NIRS.

**Supplementary Information:**

The online version contains supplementary material available at 10.1186/s13068-025-02662-1.

## Background

2,3-butanediol (2,3-BDO) is an economically important platform chemical that can be used in a variety of chemical feedstocks, liquid fuels, and biosynthetic building blocks. For example, 2,3-BDO can be dehydrated into either the fuel additive methyl ethyl ketone [1] or into 1,3-butadiene [2], which can be used as a building block for synthetic rubber or oligomerized in high yields to gasoline, diesel, and jet fuel [3, 4].

2,3-BDO can be produced efficiently by fermentation by a variety of microorganisms including *Zymomonas mobilis*, *Klebsiella* sp., *Enterobacter* sp., *Serratia* sp., *Bacillus* sp., and the yeast *Saccharomyces cerevisiae* [4–15]. Batch fermentations using an engineered *Zymomonas mobilis* produce 2,3-BDO titers of approximately 50 g/L using lignocellulosic sugars derived from corn stover; fed-batch fermentations produce 2,3-BDO titers above 120 g/L [15]*.*

*Z. mobilis* fermentations producing 2,3-BDO are more complex than corresponding ethanol fermentations [16]. A major source of complexity is that the fermentations are micro-aerophilic, meaning very low concentrations of dissolved oxygen (DO) are necessary for 2,3-BDO production due to an electron imbalance in the constructed 2,3-BDO metabolic pathway; 2,3-BDO production leads to excess NADH and NADPH generation. Because of this, small changes in DO concentration can drive the fermentation to undesired products. DO-deficient conditions result in irreversible glycerol production using a competitive metabolic pathway due to the lack of an electron acceptor, while excess DO concentrations result in acetoin rather than 2,3-BDO production in a separate metabolic pathway. Furthermore, the optimal DO concentration is not fixed, but depends on the identity and concentration of the specific sugars being utilized [4, 17] and, in fed-batch fermentations, the substrate feed rates [17]. Because of these complexities, the concentrations of DO, the substrates glucose and xylose, and the products 2,3-BDO, acetoin, and glycerol must be monitored closely to optimize 2,3-BDO production by controlling both substrate feed rate and DO concentration.

The time requirements of traditional analytical techniques prevent operators from controlling a 2,3-BDO fermentation in real time. Typically, measurements of the glucose and xylose concentrations are performed off-line, using high-performance liquid chromatography (HPLC) or automated enzymatic assays [18]. An aliquot of sample is removed from the fermentation, filtered, diluted to ensure analyte concentrations fall into the calibration range of the instrument, and then injected into the instrument of choice. As these processes can take up to 30 minutes for one sample, the actual time required after sample collection to receive actionable concentration data for multiple fermenters can be on the order of hours. Furthermore, to fully evaluate fermentation performance, understanding both substrate and product concentrations is necessary. The products 2,3-BDO, acetoin, and glycerol are typically analyzed via an additional HPLC analysis using a separate column, thereby doubling the analytical requirements.

Near-infrared spectroscopy (NIRS) is a non-destructive analytical technique that characterizes a material’s interaction with light in the near-infrared region (approximately 13,333 to 4000 cm^−1^). Overtones and combinations of fundamental vibrational modes absorb electromagnetic radiation in the near-infrared region in a broad, overlapping fashion. NIRS paired with multivariate statistical analysis (chemometrics) has been widely used as a rapid analytical tool for at-line and on-line process monitoring and control for a variety of industries, including food [19–21], agriculture [22, 23], and pharmaceutical [24–26]. Due to the presence of extreme multicollinearity in NIRS, regression techniques that include dimensionality reduction or wavelength selection are needed to build robust models. Partial Least Squares (PLS) regression [27] is a common linear regression technique used with NIRS data. Since PLS regression is a linear modeling algorithm, the resulting models have several benefits, including easier model interpretability and decreased computational and data requirements compared to more complicated non-linear model algorithms [28]. PLS modeling can provide a useful workflow for the rapid prototyping and deployment of NIRS-based predictive models during the research and development phase of a conversion process.

NIRS has been used to monitor 2,3-butanediol in wine samples [29], glycerol in process samples for the transesterification of vegetable oil for biodiesel production [30], acetoin in wine samples [31], and glucose and xylose in aqueous fermentation matrixes [32–34]. However, there are no previous literature examples of the simultaneous monitoring of all five of these constituents in a single matrix. NIRS has been successfully developed and implemented as a fermentation monitoring tool to measure substrate and product concentrations in multiple cases [32, 35–42].

To the extent of the authors’ knowledge, there are no previous literature examples of using NIRS for process monitoring and control of any 2,3-BDO fermentations. While NIRS has been used to identify fermentation phase changes via glucose concentration monitoring in mammalian cell cultivations [33], using NIRS to address the unique challenges of this *Z. mobilis* fermentation (producing a micro-aerophilic condition and balancing a redox reaction) is a novel application of the technique.

**Fig. 1:**
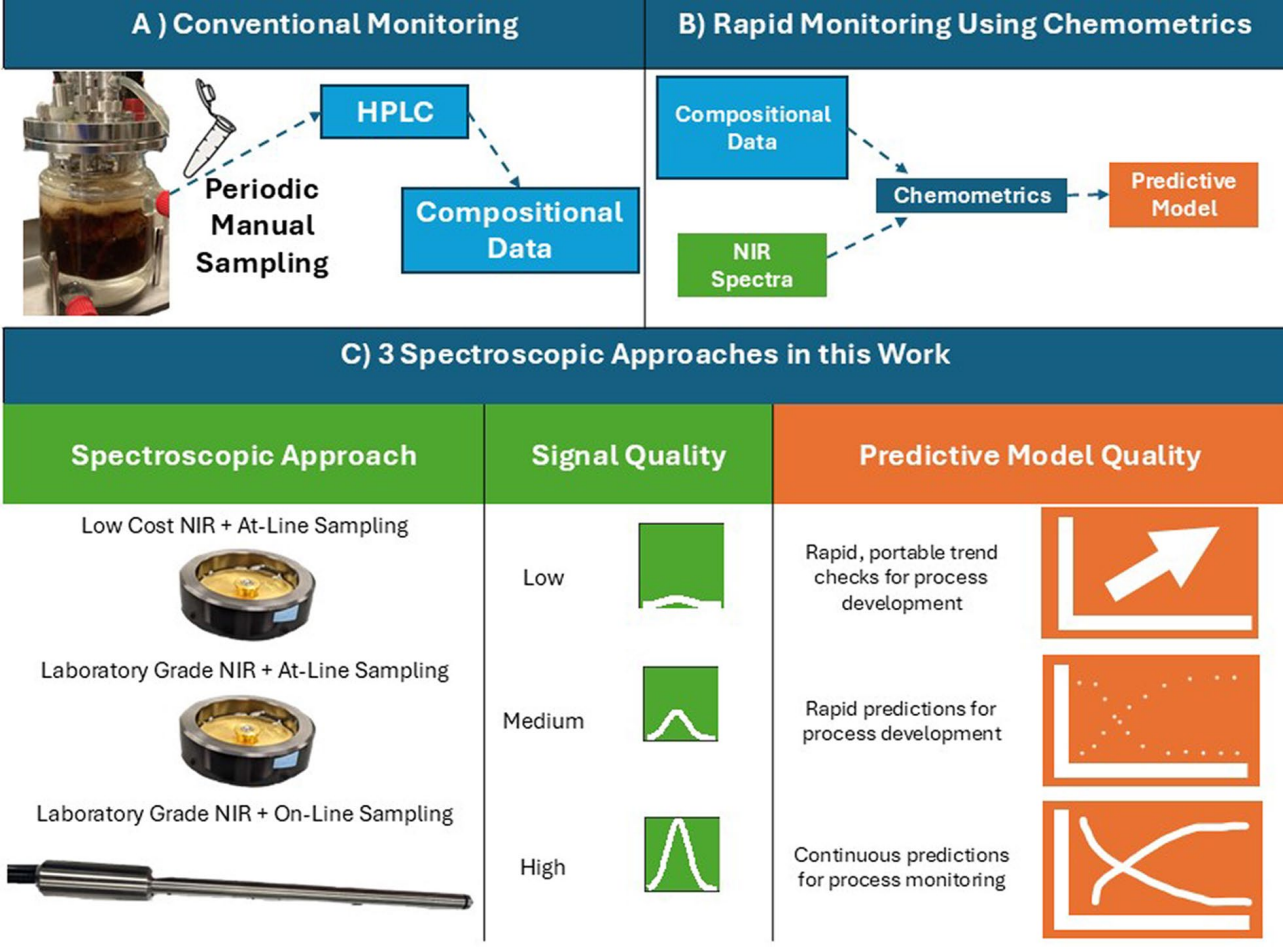
Overview of experimental work. **A** Conventionally, bioreactors are sampled periodically; samples are filtered and analyzed via HPLC to produce compositional data that indicates how well the fermentation ran. **B** Conventionally collected compositional data are combined with near-infrared (NIR) spectra to produce partial least squares PLS predictive models useful for real-time monitoring. **C** Comparison of the three different spectroscopic approaches evaluated in this work, depicting the spectroscopic approach (grade of spectroscopy and sampling method), quality of signal produced, and quality of resulting predictive model

In this work, we evaluate multiple instruments and sensing modality combinations for producing NIRS/chemometrics-based solutions for rapid monitoring and process control of a novel *Z. mobilis* 2,3-BDO fermentation (Fig. 1). We believe this work provides a framework or approach to use during the evaluation of NIRS/chemometrics as an analytical tool for fermentation process monitoring. First, we demonstrate the utility of preliminary feasibility testing with pure compounds to identify whether a fermentation has potential for monitoring via NIRS. We then provide a thorough comparison of the potential of at-line monitoring of real fermentations for these compounds by comparing two different NIRS spectrometers (one laboratory-grade instrument, and one low-cost instrument with reduced spectral range and sensitivity) showing the impacts of analyte signal strength on model performance. We then demonstrate how sampling can be eliminated from a NIRS fermentation monitoring scheme using on-line monitoring with fiber optic probes. Finally, we evaluate how an on-line model built for one fermenter transfers to a different fermenter with a 20-fold larger volume to demonstrate the use of NIRS/chemometrics in scale-up.

## Materials and methods

### Laboratory fermentation sample collection

Samples were obtained from a variety of bench-scale fermentation experiments performed with the goal of optimizing the conversion of mixed sugars to 2,3-BDO using genetically engineered strains of *Z. mobilis.* Most experiments used a mixture of glucose and xylose at a roughly 2:1 ratio, although some experiments also included small amounts of arabinose, and some contained only glucose. Real cellulosic hydrolysates typically included all three sugars, although some fermentations were tested using both pure sugars and lignocellulosic sugars from corn stover in a background medium of 10 g/L yeast extract and 2 g/L KH_2_PO_4_. Strain development work that occurred during the campaign resulted in the use of 3 unique stable strains of *Z. mobilis*, each possessing slightly different fermentation performance.

Fermentations were performed in multiple reactor configurations, including laboratory shaker flask experiments (50–200 mL), laboratory fermenter experiments (500 mL Sartorius BioStat Q-plus and 10L Eppendorf BioFlo 230), and pilot-scale fermenters (100–1500L). For fermenter experiments, the DO concentration was influenced by the aeration setpoint and the agitation speed. An aeration setpoint of 0.2 volume per volume per minute (VVM) was used in all fermenters. The agitation speed varied with different vessel sizes, and was chosen to achieve an oxygen mass transfer coefficient (*k*_*L*_*a*) of approximately 20 hr^−1^, previously determined in water using the dynamic method for estimating *k*_*L*_*a* [43]. During the fed-batch portion of the fermentation, the sugar feed rate was adjusted based on *Z. mobilis’* glucose consumption rate calculated from the residual glucose in the fermentation broth.

A total of 194 unique fermentation experiments were sampled resulting in a total of 1332 unique samples. For this work, we define a unique experiment as a fermentation performed in a single vessel from the time of inoculation to the time the fermentation was ended.

### Primary analytical methods

Primary analytical data were generated using HPLC. The concentrations of glucose and xylose were quantified using NREL Laboratory Analytical Procedures [44] for monomeric sugar composition. The concentrations of 2,3-BDO, acetoin, and glycerol were measured by injecting 6 µL of 0.02 µm filtered liquor onto an Agilent 1100 series system equipped with an Aminex HPX-87H column and a cation H^+^ guard cartridge (Bio-Rad Laboratories) at 55 °C with a mobile phase of 0.01 N sulfuric acid at a flow rate of 0.6 mL/min and a refractive index detector (RID). The total concentration of 2,3-BDO was measured as the sum of meso-2,3-butanediol and (2S,3S)-butanediol. All HPLC calibration standards were acquired from Absolute Standards (Hamden, CT).

### NIRS spectra collection

Spectra were collected using two different instruments and two different sampling modalities.

The laboratory-grade instrument was a Thermo Antaris II FT-NIRS spectrometer (Thermo-Fisher) operated using Thermo RESULT and OMNIC software. As the name implies, the Thermo Antaris spectrometer is a Fourier Transform instrument and uses a Michelson interferometer, so its native spectral unit is frequency. For each spectrum, 128 scans were averaged per sample across a wavenumber range of 12,003 to 3772 cm^−1^ for at-line measurements and 9503 to 4000 cm^−1^ for on-line measurements (8 cm^−1^ resolution). An internal gold reference was used for reference measurements. It took approximately two minutes to collect each spectrum.

The low-cost instrument was a NIRONE S2.5 NIRS spectrometer (Spectral Engines) operated using proprietary software provided by Spectral Engines. The NIRONE spectrometer uses a micro-electromechanical (MEMS) Fabry-Perot interferometer, and its native spectral unit is wavelength. Each spectrum was collected over a range of 2000 to 2450 nm (1 nm resolution). 5000-point measurements were averaged at each wavelength, and the lamp intensity was set to 100%. A white reference target was used for reference measurements, and an empty quartz cup with gold transflectance backing was used for dark measurements. It took approximately 4 min to collect each spectrum.

Transflectance spectra were collected for all feasibility samples and at-line samples for both instruments. At-line samples were filtered through a 0.02 µm nylon filter to remove insoluble solids. Approximately 0.1 ml of filtered sample was placed in an optical-grade quartz ring cup fit with a 0.2 mm pathlength gold transflectance plate prior to spectra collection. Figure 1C displays the ring cup and gold transflectance plate used.

On-line spectra were collected using one of two fiber optic probes inserted into the fermentation vessel and connected to the laboratory-grade instrument. Figure 1C displays one of two the fiber optic probes used in this work. The first fiber optic probe, used for 500 mL fermentation experiments, was a 2-m-long, 2 × 600 µm broadband 0.22 NA fiber optic transflectance dip probe fit with a 2 mm pathlength tip (Avantes USA). The probe was submerged within the fermentation vessel to sit below the aeration impellers with the optical slit facing toward the center of the vessel to allow fermentation broth to easily move through the slit during aeration. The probe had a 304 stainless steel probe shaft that was 100 mm in length with an outer diameter of 6.6 mm. It was connected to the laboratory-grade NIRS instrument via an SMA-905 adapter. Spectra were collected continuously from inoculation to the end of aeration. Reference spectra (using the internal gold reference) were taken between each sample collection to correct for instrument drift. The second fiber optic probe was used to test the transferability of the on-line chemometric model to a larger volume fermenter. To accommodate the larger fermenter volume, a custom fiber optic probe was used—a 2.5-m-long, 2 × 600 µm broadband 0.22 NA fiber optic transflectance dip probe fit with a 6.4 mm OD 2 mm pathlength tip (Avantes USA). This probe featured a 405 mm in length × 12 mm OD 304 stainless steel probe shaft to accommodate the larger bioreactor. As with the 500 mL on-line configuration, the fiber optic probe connected to the laboratory-grade NIRS spectrometer via an SMA-905 adapter. Spectra were collected continuously from inoculation to the end of the experiment. Reference spectra (using the internal gold reference) were taken between each sample collection to correct for instrument drift.

### Feasibility testing spectral dataset

To test the feasibility of NIR/chemometrics for monitoring the constituents of interest in this fermentation, we used the laboratory-grade NIRS spectrometer in transflectance mode as described above to first collect spectra of prepared solutions of each substrate (glucose and xylose at 150 g/L each) and product (2,3-BDO at 150 g/L and acetoin and glycerol at 100 g/L each) separately. We then collected spectra on a small population (*n* = 40) of mixtures containing randomly distributed, uncorrelated concentrations of all five constituents at concentrations ranging from 0 to 60 g/L. Constituent concentrations were verified via HPLC.

### Spectral datasets used in this work

A total of five different datasets were generated and used in this work:

1. A laboratory-grade feasibility dataset consisting of spectra collected from mock constituent samples using the laboratory-grade spectrometer and a transflectance cell.
2. A laboratory-grade at-line dataset consisting of spectra collected from filtered fermentation samples using the laboratory-grade spectrometer and a transflectance cell.
3. A low-cost at-line dataset consisting of spectra collected from filtered fermentation samples using the low-cost spectrometer and a transflectance cell.
4. A laboratory-grade on-line dataset consisting of spectra collected from laboratory fermentations in 500 mL fermenters using a fiber optic probe.
5. A scale-up on-line dataset consisting of spectra from a single laboratory fermentation experiment in a 10L fermenter using a fiber optic probe.

### Multivariate analysis

We used the open-source programming language R [45] for all modeling and statistical analysis. For spectral transformation and calibration population selection, we used the *prospectr* package [46]. To build and cross-validate PLS models, we used the *pls* package [47] using the orthogonal scores algorithm for model fitting. All data cleaning, wrangling, and visualization was done using the *tidyverse* collection of packages [48], the package *openxlsx* for reading data [49], and the *ggplot* extension packages *egg* [50], *ggtext* [51], *plotly* [52], and *patchwork* [53] for visualizations.

The R scripts used for spectral transformation and regression modeling can be found on the NREL github repository.

### Spectral transformation

All spectra collected using the laboratory-grade spectrometer were truncated to 9000 to 4000 cm^−1^ to remove very noisy portions of the spectra with demonstrated low analyte signal. No portions of the spectra collected using the low-cost spectrometer were removed. We then used the Standard Normal Variate (SNV) transformation to correct for light scatter on all spectra, followed by Savitzky–Golay filtering (SG) (using the 2nd order polynomial and 1 st derivative smoothing). The selected smoothing window was evaluated over a range of 3 to 21 for each dataset and selected based on model performance. Prior to model fitting, each calibration set was mean centered.

After SG smoothing, the collected spectra were truncated again to remove non-informative regions. This truncation is discussed in more detail below.

### Spectral noise analysis

**Fig. 2:**
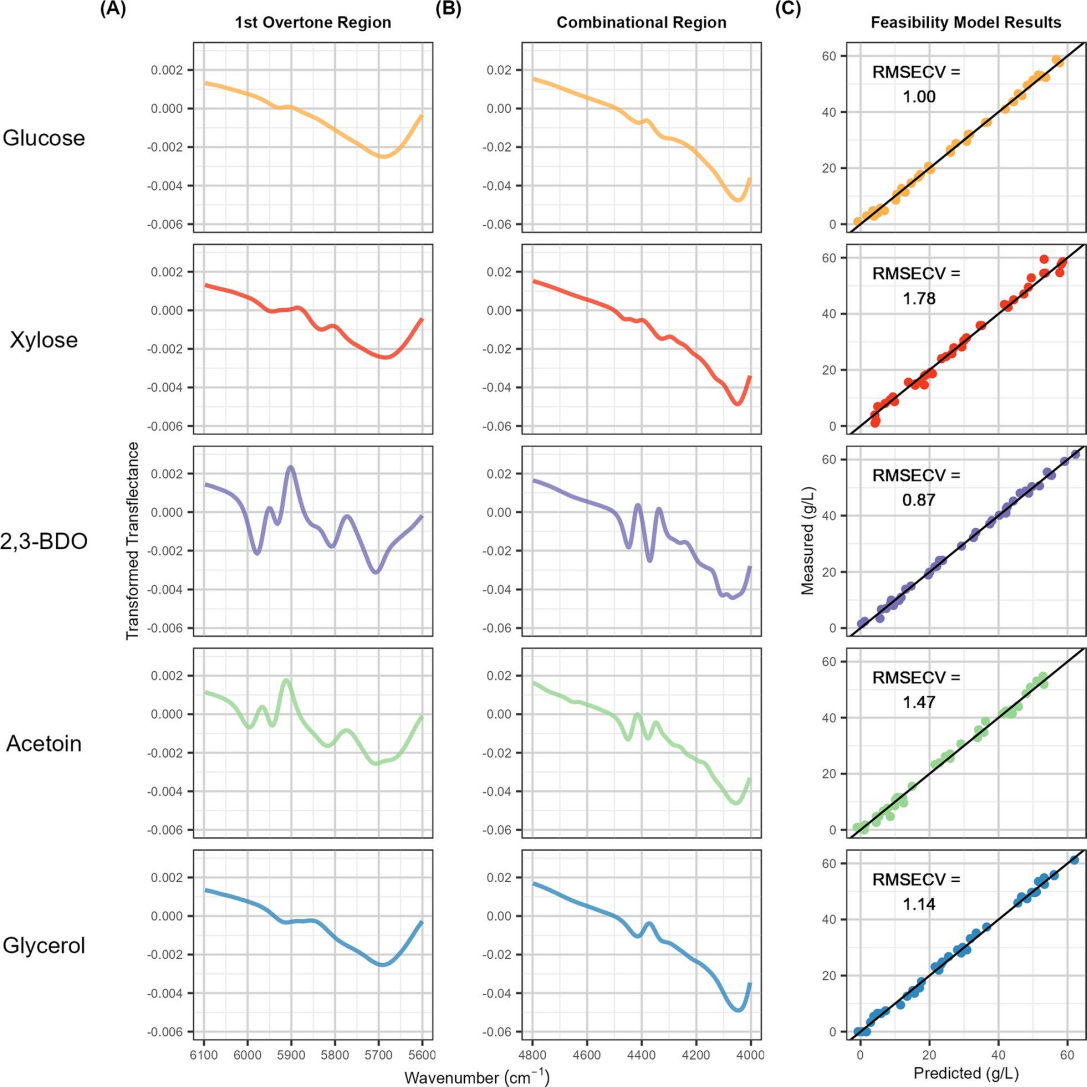
Standard constituent spectral signatures and feasibility study results. Transformed transflectance spectra (zoomed in to regions of high signal) for aqueous standards of (top to bottom) 150 g/L glucose, 150 g/L xylose, 150 g/L 2,3-BDO, 100 g/L acetoin, and 100 g/L glycerol collected using the laboratory-grade at-line data collection method. **A** The 1 st overtone region of the spectra from 6100 to 5600 wavenumbers (cm^−1^). C–H 1 st overtone bands associated with the unique C–H bonding environments of each standard are observed [54]. **B** The combinational region of the spectra from 4800 to 4000 cm^−1^. O–H combination (O–H stretch/C–O stretch) and C–H combination bands associated with the unique bonding environments present in each molecule are observed [54]. **C** Leave-One-Out Cross-Validation (LOOCV) performance results for laboratory-grade, at-line model produced from 40 samples of random, uncorrelated concentrations of the 5 constituents of interest. Root Mean Squared Error of Cross-Validation (RMSECV) for each constituent is shown in the top left corner of each plot

We use spectral noise analysis (using a similar approach as used in reference 47) to quantify differences in instrument noise between the low-cost and laboratory-grade instruments. Spectra of nanopure water were collected throughout the scanning campaign using the at-line configuration (*n* = 20) and processed using the same signal processing techniques and selected parameters used for final modeling. To account for differences in spectral resolution, we subsampled 10 evenly spaced wavenumbers between 4480 and 4300 wavenumbers (the area in the combination range most associated with constituent signal, as observed in Fig. 2B). The mean standard deviation of the processed sample spectra across the selected wavenumbers was calculated for both instruments and compared.

### Calibration, validation, and unique sample sets

We divided each of the three experimental datasets into three subsets. First, one unique experiment that was representative of a typical fermentation experiment was removed and labeled a unique validation set. Because each fermentation experiment has unique variability associated with it (e.g., growth rate, temperature fluctuation, aeration, vessel volume, media concentration, feed rates), all samples from a single experiment are correlated in some way. Holding all the samples from a single experiment out as a unique validation set provides an estimate of overall model performance that is independent of inter-experiment correlations, but exhibits some bias, as it is not fully representative of the variability in the calibration set.

The remaining samples were then divided into calibration and independent validation sets using the Kennard–Stone algorithm on the PCA scores that explained 95% of the total transformed spectral variance. For the laboratory-grade at-line dataset, 40% of the samples were held out for independent validation. For both the low-cost at-line and laboratory-grade on-line datasets (which were substantially smaller than the laboratory-grade at-line dataset), 25% of the samples were held out for independent validation. Independent validation tests the model’s prediction capability across the full variability of the calibration set without the risk of bias.

### Model validation

Leave-one-out (LOO) cross-validation was used to tune each model to the appropriate number of principal components (PCs). Models were evaluated across an initial range of 1 to 20 PCs. To avoid overfitting a given outcome of interest, each constituent was evaluated individually for the number of components necessary to fully explain the relationship between spectra and primary analytical measurements.

We evaluated model performance by comparing the root mean squared errors (RMSE) associated with the predictions of the calibration set (RMSEC), the cross-validated models (RMSECV), the independent validation set (RMSEP), and the unique experiment set (RMSEP_unq_). The nature of fermentation optimization led to sugar substrates and the desired product having higher concentration ranges than the undesired products, and to each of the three datasets having slightly different ranges of the constituents of interest. To allow for comparison among constituents in a given dataset, as well as among different datasets, we normalized the RMSE calculations to the range of each constituent in each calibration set for each model, e.g., the RMSECV, RMSEP, and RMSEP_unq_ values for the laboratory-grade at-line glucose prediction model were normalized by the difference between the maximum and minimum glucose concentrations in the laboratory-grade at-line calibration set.

We tested for heteroscedasticity in each validation, cross-validation, and independent validation set by visually evaluating the predicted vs. residual plots for fanning or funneling.

## Results and discussion

### Feasibility test results

Aqueous spectra were collected from standards of all five constituents using that laboratory-grade at-line configuration. Glucose, xylose, and 2,3-BDO were evaluated at 150 g/L, while acetoin and glycerol (undesirable coproducts with expected lower concentrations) were evaluated at 100 g/L. Figure 2 shows the transformed transflectance signal responses from all constituents in (A) the 1 st overtone region from 6200-5600 cm^−1^ and (B) the combinational region from 4800 to 4000 cm^−1^. All constituents produced a unique NIRS signal, indicating identification of each constituent was possible via NIRS/Chemometrics. Two important observations are presented in Fig. 2A and B. First, the signal intensity in the combinational region is much stronger than in the 1 st overtone region (note the 10-fold y-axis scale difference between the two plots). Second, the spectral signatures of 2,3-BDO and acetoin are substantially stronger than those of glucose and xylose.

Standards were mixed to produce aqueous matrices of uncorrelated concentrations of all constituents in the range 0–60 g/L, and PLS-2 modeling was performed to test the feasibility at NIRS/chemometrics for quantifying mixtures of these five constituents. Figure 2C shows the cross-validation performance results of PLS-2 models produced, including the RMSECV value. The mock models performed very well with RMSECV values between 0.87 and 1.78 g/L for all constituents. Residual plots showed no heteroscedasticity, indicating that linear regression modeling with at-line NIRS would be a reasonable method for quantifying mixtures of glucose, xylose, 2,3-BDO, acetoin, and glycerol at concentration ranges between 0 and 60 g/L.

### Primary analytical data distributions

The laboratory-grade at-line dataset is by far the most extensive dataset, containing 700 samples taken from 109 unique fermentation experiments across the development of 3 stable strains of *Z. mobilis*. The low-cost dataset contains 363 samples from 62 unique experiments, and the laboratory-grade on-line dataset contains 112 samples from 20 unique experiments. Across the entire campaign, fermentation experiments were occasionally contaminated by bacteria that converted sugar into lactic acid, ethanol, and acetic acid. We excluded a small number of samples with concentrations of ethanol, lactic acid, or acetic acid greater than 2.5 g/L. We did this for two reasons. First, we determined that the contaminated samples behaved as outliers and skewed model performance when included. Furthermore, because such contaminations were rare and typically caught early by the researchers performing the fermentations, there were not enough contaminated samples to produce accurate modeling of the spectral variability associated with contamination.

All product distributions (2,3-BDO, acetoin, and glycerol) exhibit positive skew. 2,3-BDO exhibits a positive skew due to the nature of research and development—the optimization of high titer yields took a lot lower titer yields to achieve, leading to a dataset with lots of low and moderate 2,3-BDO titers, and a few high titers. The positive skew for acetoin and glycerol datasets exists because the fermentations were run to optimize 2,3-BDO; when irreversibly high titers of glycerol and acetoin were noticed in experiments, those experiments were ended early to maximize time and resources devoted to successful fermentations. Data distributions for glucose, xylose, 2,3-BDO, acetoin, and glycerol concentrations are shown in Supplemental Table S1 and Supplemental Fig. S1.

In all three datasets, there are substantially more samples with low glucose concentration than with high glucose concentration, whereas the xylose concentration data are more evenly distributed (i.e., the glucose concentration data exhibit a substantially more positive skew than the xylose concentration data). This difference is due to the intrinsic metabolism of the organism and the way in which experiments were performed. Only six-carbon sugars can be metabolized by wild type Z. *mobilis*; genetic modification of the wild type is necessary for the organism to utilize xylose [15, 16]. In the presence of both glucose and xylose, *Z. mobilis* will preferentially metabolize glucose. Furthermore, glucose uptake by *Z. mobilis* is more rapid than xylose*.* Finally, the majority of these data came from fed-batch fermentations, leading to generally lower substrate concentrations than batch experiments.

Individual fermentations were run on either feedstock hydrolysate or pure sugar. The matrix of cellulosic sugar hydrolysates is very different than pure sugar solutions, but these matrix effects appear not to affect the PLS models. Supplemental Fig. S3 through S9 show predicted vs. measured results for all models, colored by starting material type. We saw no clear differences in the distribution of the variance of the residuals between the two sugar sources; the multivariate modeling approach we used was robust against sugar source differences in the sample matrices.

Some intrinsic relationships exist among these constituents. For example, the substrates glucose and xylose have positive correlations with each other in all datasets. Glucose exhibits a negative correlation with all products, while xylose generally exhibits a weaker negative correlation with all products.

The undesired coproducts glycerol and acetoin show different correlations with the product 2,3-BDO. As mentioned above, the conversion of sugars into glycerol occurs via a different metabolic pathway than 2,3-BDO [4, 17]. Since both glycerol and 2,3-BDO are irreversible end products to the conversion pathway, both have positive correlations with fermentation time and therefore to each other. In contrast, acetoin is an intermediate in the 2,3-BDO pathway and its concentration is therefore less strongly correlated fermentation time and, therefore, to 2,3-BDO concentration. Supplemental Table S2 displays the correlation coefficients among the major constituents in each dataset, while Supplemental Fig. S2 provides pairs plots for visualization of the correlations.

### Spectral datasets

**Fig. 3:**
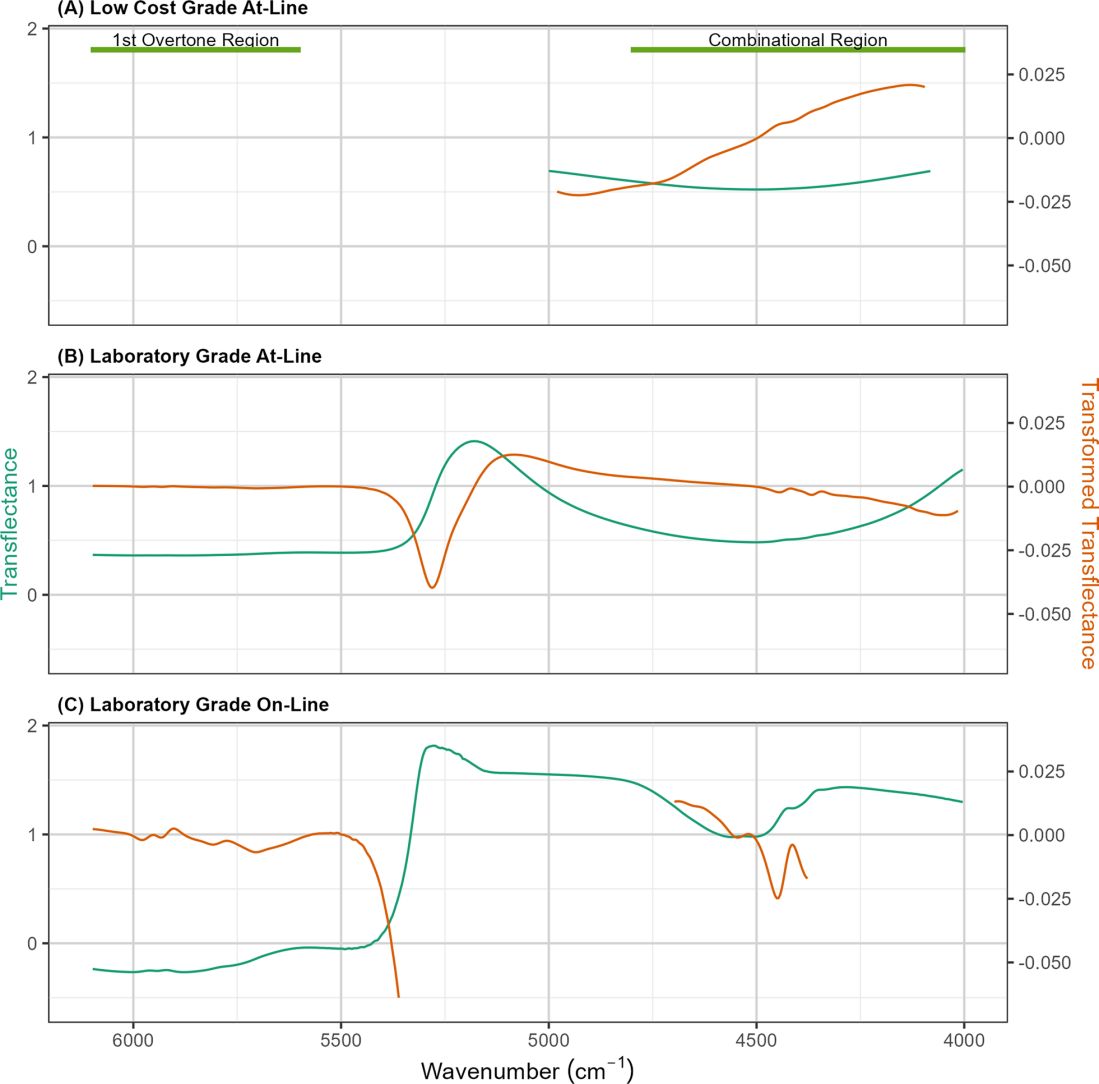
NIRS spectra from at-line and on-line datasets. Average Near-infrared (NIR) transflectance spectra (green) and transformed transflectance spectra (orange) of filtered BDO fermentation liquors collected across varying time points and fermentation parameters comprising the (**A**) low-cost at-line dataset, **B** laboratory-grade at-line dataset, and **C** laboratory-grade on-line dataset. The differences in the spectral ranges of the three instrument modalities are due to differences in the intrinsic spectral ranges of the two instruments (plot **A** vs. plot **B**) and the use of a fiber optic probe (plot **B** vs. plot **C**). For instance, there is substantially less useful signal in the combinational range (~4500 cm^−1^) in the spectra collected using the low-cost instrument than the lab-grade instrument, despite the pathlength of signal being the same. Furthermore, there is substantially more useful signal in both the combinational and 1^st^ overtone regions in the spectra collected using the on-line configuration, which is likely due to the pathlength of transflectance being 10× greater (0.2 mm to 2 mm)

Figure 3 shows the average raw and transformed transflectance spectra for each model dataset. The spectra from the three different spectroscopy modalities look substantially different despite similar concentration profiles in each dataset. All three modalities show clear spectral signals in the combinational range that correspond to the spectral signatures of the pure-component spectra in Fig. 2. The spectral signatures in the 1 st overtone region are more difficult to distinguish because (as observed in the standards) the overall signal intensity of the combinational region is much higher than that of the 1 st overtone region.

The spectral range and the absolute signal intensity of the average low-cost at-line spectra in Fig. 3A are substantially smaller than the mean laboratory-grade at-line spectra in Fig. 3B despite using identical sample presentation. After controlling for differences in resolution and range, spectral noise analysis revealed that the average noise in water spectra from the low-cost instrument was 4.8× greater than that observed from the laboratory-grade instrument (Supplemental Fig. S11). Lower signal response, greater noise, and decreased range are necessary compromises in the development of low-cost instrumentation that can limit model performance.

The average spectra of the at-line (Fig. 3B) and on-line laboratory-grade spectrometers (Fig. 3C) look substantially different from each other. This is due to the differences in the physics of sample presentation. Absorbance readings were saturated in the on-line dataset at a characteristic water band signal—the region between 5400 and 4800 cm^−1^—as well as in the region below 4300 cm^−1^. Two factors contribute to the saturation: (1) the much longer pathlength in the on-line spectral dataset (2.0 mm vs. 0.2 mm) results in much higher overall absorption, and (2) light absorption by the 2 m fiber optic probe results in substantial attenuation of the entire on-line spectra. The saturated regions were removed from the calibration prior to transformation and modeling. The on-line laboratory-grade dataset shows a greater signal-to-noise ratio for the regions associated with constituents of interest than the at-line laboratory-grade dataset in both the combinational and 1 st overtone regions. This is primarily due to the longer pathlength, which produces greater analyte signal despite the additional complexity of the fiber optic probe and the sample matrix effects from the unfiltered fermentation broths (recall that at-line fermentation broth samples were filtered prior to scanning).

### At-line model predictions

**Fig. 4:**
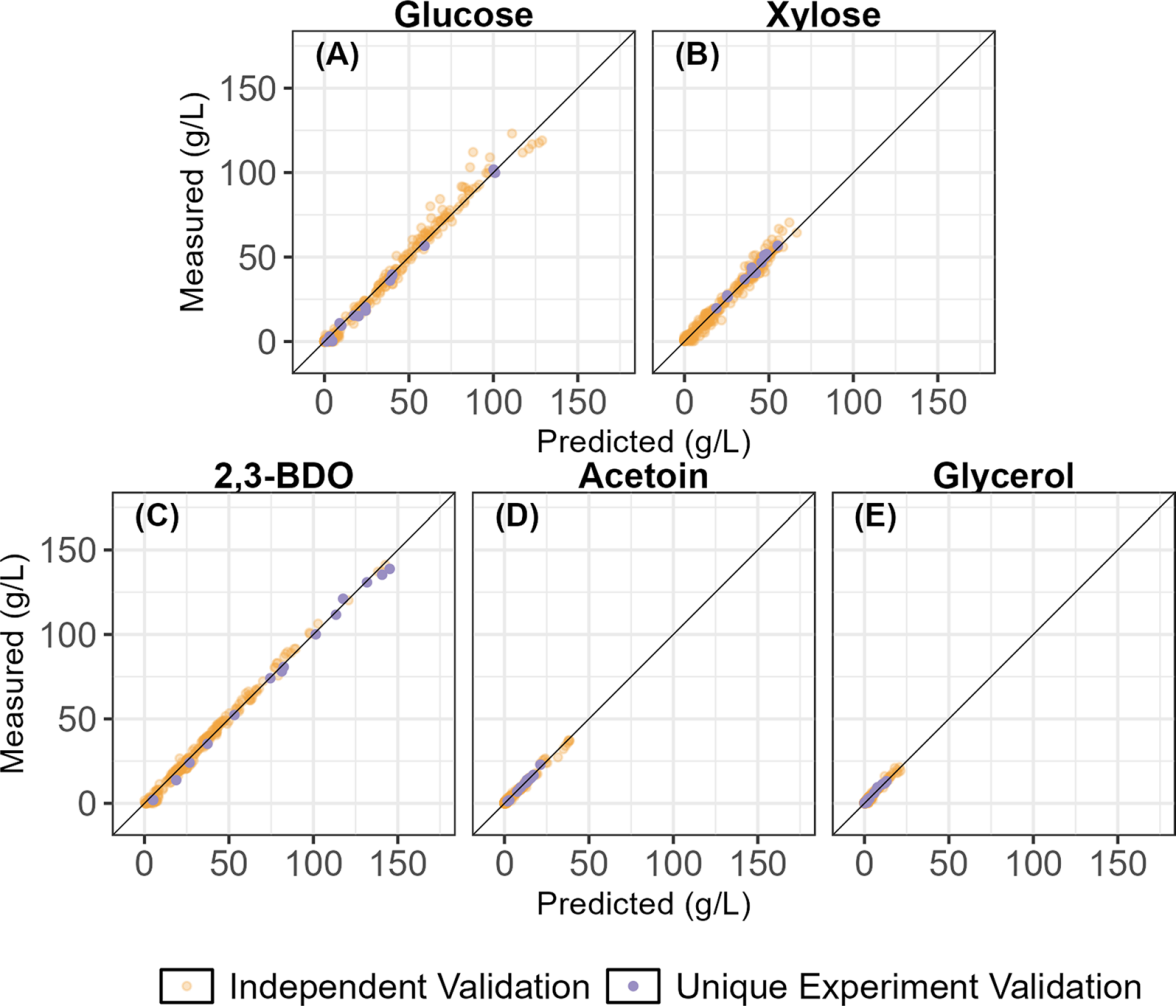
Accuracy of laboratory-grade at-line prediction model. Measured versus predicted independent validation results for the lab-grade at-line dataset. Concentrations are reported in units of grams per Liter (g/L). In these experiments, *Zymomonas mobilis* converts glucose (**A**) and xylose (**B**) to 2,3-BDO (**C**) with undesirable coproducts acetoin (**D**) and glycerol (**E**). Independent validation samples are depicted in orange. This dataset was split to hold out 40% of the samples for independent validation using the Kennard–Stone algorithm performed on the PCA scores of the transformed spectra. Unique experiment validation samples are depicted in purple. No samples from this experiment were used to train the model, and thus, they represent the model’s ability to predict the composition of samples from a completely independent dataset. The undesired coproducts acetoin and glycerol have substantially smaller concentration ranges than the substrates glucose and xylose and the desired product 2,3-butanediol. Some heteroscedasticity is seen in glucose predictions above 95 g/L, which could be explained in part by high dilution error in the primary HPLC measurements.

The goal of developing a rapid at-line prediction model for 2,3-BDO production was to provide measurements of constituents with low enough error that the rates of substrate uptake and product formation could be accurately measured, allowing for real-time control of aeration and substrate feeding to maximize 2,3-BDO production. Figure 4 shows the laboratory-grade at-line NIRS model predicted versus HPLC measured concentration values for all measured components using the independent validation dataset (orange) and the unique experiment validation dataset (purple). The first column of Supplemental Table S3 shows the performance parameters for all constituents. Root mean squared errors of prediction for all constituents using the laboratory-grade at-line model were less than 4 g/L. To provide context, the primary analytical method, HPLC, can provide approximately tenfold better accuracy for the five constituents. Given the propagation of uncertainty in developing a secondary analytical method with NIRS/chemometrics, the increase in error relative to the primary analytical method is expected. Non-linearity can be observed in glucose predictions above 80 g/L. We believe this could be attributed to compounding effects of dilution. The HPLC method used for analyzing glucose and xylose in this work has a calibration range of 6 to 36 g/L. At concentrations of glucose above 80 g/L, at least a 3× dilution is needed to bring samples into this calibration range, which introduces a compounding of human error in measurement.

**Fig. 5:**
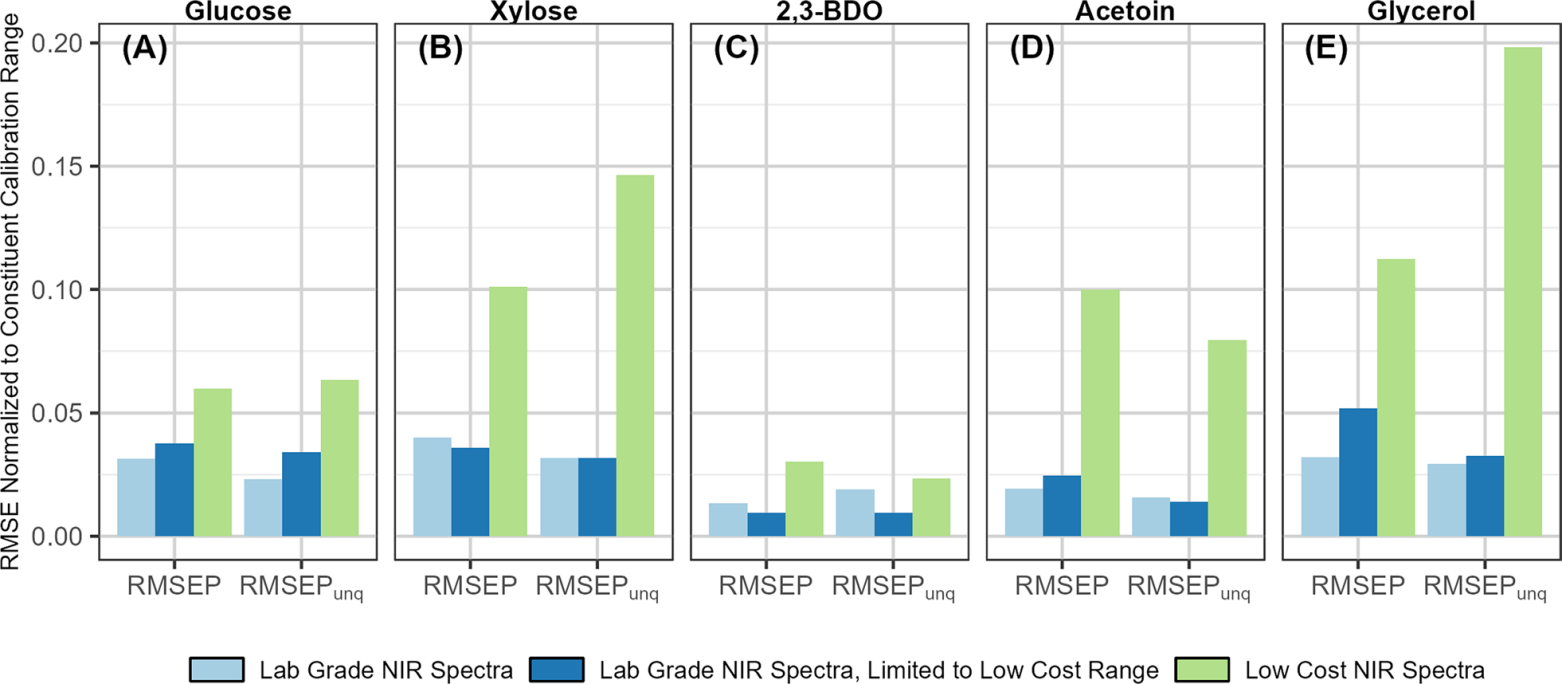
Performance comparison of at-line models. Bar chart showing model performance as measured by normalized RMSE values (RMSE divided by calibration range) by constituent for the validation sets of the lab-grade at-line model (light blue); lab-grade at-line model using spectra truncated to the low-cost spectrometer spectral range (dark blue), and the model using at-line spectra from the low-cost spectrometer (light green) for substrates glucose **A** and xylose **B**, product 2,3-BDO **C**, and undesirable coproducts acetoin **D** and glycerol **E**. RMSEP is the root mean squared error of prediction for the independent but spectrally representative validation sample set. RMSEP_unq_ is the RMSEP for the unique experiment validation sample set. The lab-grade NIRS instrument spectra result in models with better performance (e.g., lower normalized RMSEP) than the low-cost NIRS instrument even when the spectral range is constrained. The total BDO models show the best performance across all spectroscopy modalities.

Figure 5 shows the comparison of model performance as measured by normalized RMSE values for the independent validation and unique experiment datasets for the three at-line datasets: the laboratory-grade, the laboratory-grade using the same spectral range as the low-cost instrument, and the low-cost, each normalized by the range of the constituent in the calibration dataset. Additional data on the model performance parameters are provided in Supplemental Table S4.

Both models using laboratory-grade spectra were superior to the model developed using low-cost spectra even when the spectral range of the laboratory-grade dataset was truncated to match the low-cost range, indicating that the lower signal strength and increased noise of the low-cost spectrometer limit its performance. Using the low-cost spectral range with the laboratory-grade instrument decreased model performance for glucose and glycerol prediction, indicating that the missing 1 st overtone region provides additional information for monitoring these constituents. Conversely, using the low-cost spectral range increased model performance for 2,3-BDO prediction performance, indicating that the combinational range provides a more practically useful signal for monitoring 2,3-BDO in this matrix than the full spectra.

### On-line model predictions

The on-line dataset showed decreased model performance as compared to either of the at-line laboratory-grade models across all constituents as measured by RMSE and correlation coefficient values. This is unsurprising because despite using the same optical bench as the at-line configurations, the on-line model has several differences. First, the calibration set for the on-line model is substantially smaller than either of the at-line calibration sets. Second, light attenuation through the fiber optic cable decreases overall signal strength. Third, the constituent signals are confounded by suspended cell mass in the fermenter. Finally, while the longer pathlength increases signal intensity, it also exacerbates signal saturation due to water.

**Fig. 6:**
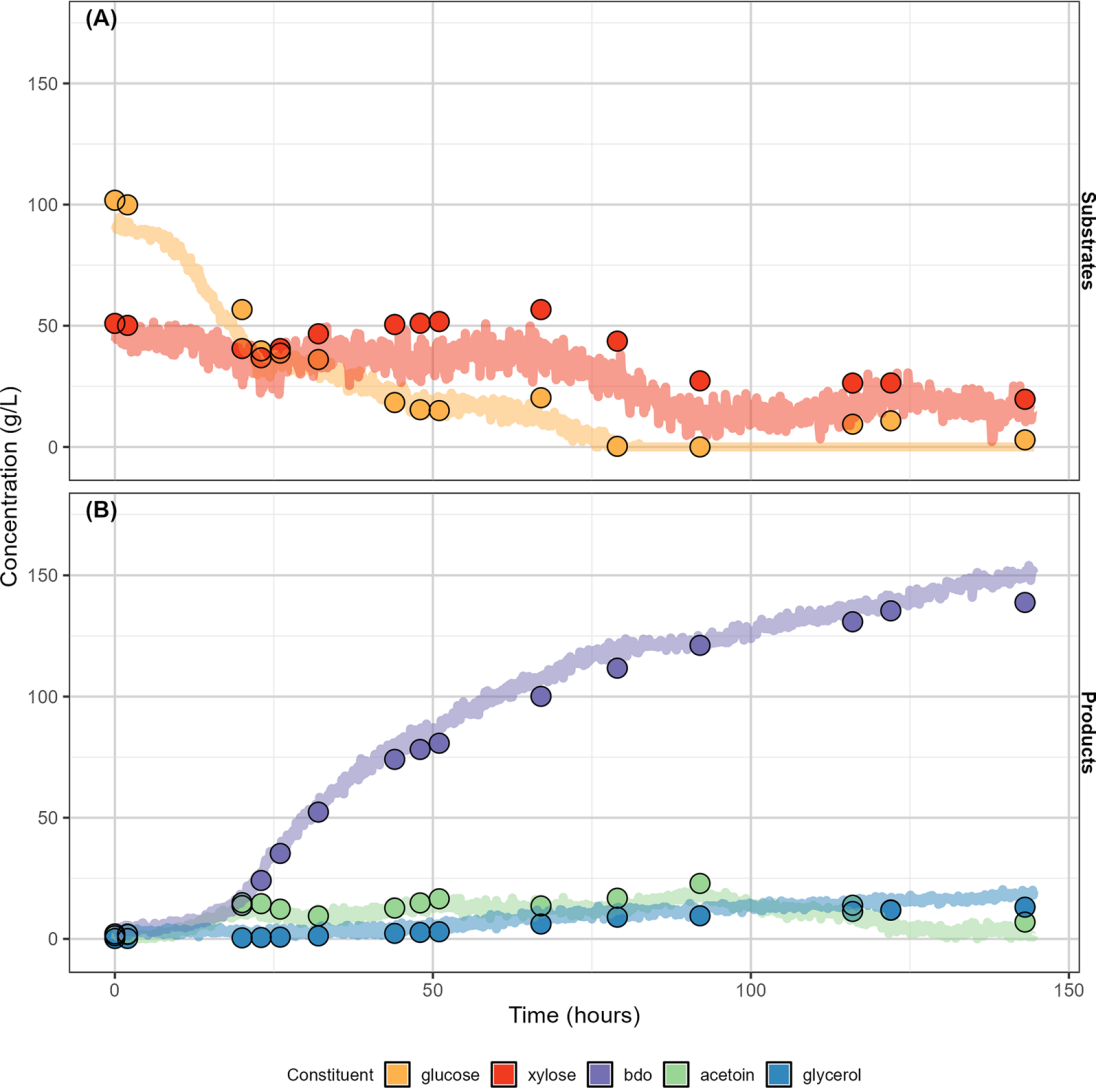
Performance of on-line calibration models—fermentation time course of a unique 500 mL fermentation. Measured (points) and predicted (line) concentrations (g/L) of fermentation substrates (**A**) and products (**B**) for a unique 500 ml fermentation experiment not used for the calibration or validation of the models. Spectra were collected on-line in a 500 mL fermenter using a 2 m-long, 2 × 600 µm broadband 0.22 NA fiber optic transflectance dip probe fit with a 2 mm pathlength tip (Avantes USA). The probe was submerged within the fermentation vessel to sit below the aeration impellers and was connected to the laboratory-grade NIRS instrument via an SMA-905 adapter. Spectra were collected continuously from inoculation to the end of aeration. Reference spectra (using the internal gold reference) were taken between each sample collection to correct for instrument drift

Nonetheless, the on-line model provides useful, actionable information. Figure 6 shows the comparison of the on-line NIRS predictions of the substrates and products of a unique *Z. mobilis* fermentation fed using pure glucose and xylose to off-line HPLC measurements. The fermentation volume (500 mL) matched that of fermentations used for calibration of the on-line model. Continuous on-line monitoring of trends in both substrate and product titers can be predicted by the laboratory-grade on-line model with sufficient accuracy to enable fermentation scientists to make real-time interventions. The predictions of the sugar substrate concentrations are not as accurate as those for the product concentrations—in some cases more than 100% difference between predicted and measured concentrations at the lowest concentrations. As mentioned previously, the spectral signatures of glucose and xylose are quite similar to each other and much weaker than the corresponding product spectra (Fig. 2), likely causing the reduced accuracy of the sugar concentration predictions. However, since the predictions of the product concentrations have very small errors, and the overall trends in the sugar concentration profiles are correct, this model could provide valuable information to operators. The limits of the model’s utility are further tested when the model is applied during scale-up experiments—an important part of fermentation process development, where the value of process monitoring increases because of the additional experimental complexity and required labor associated with scale-up experiments.

**Fig. 7:**
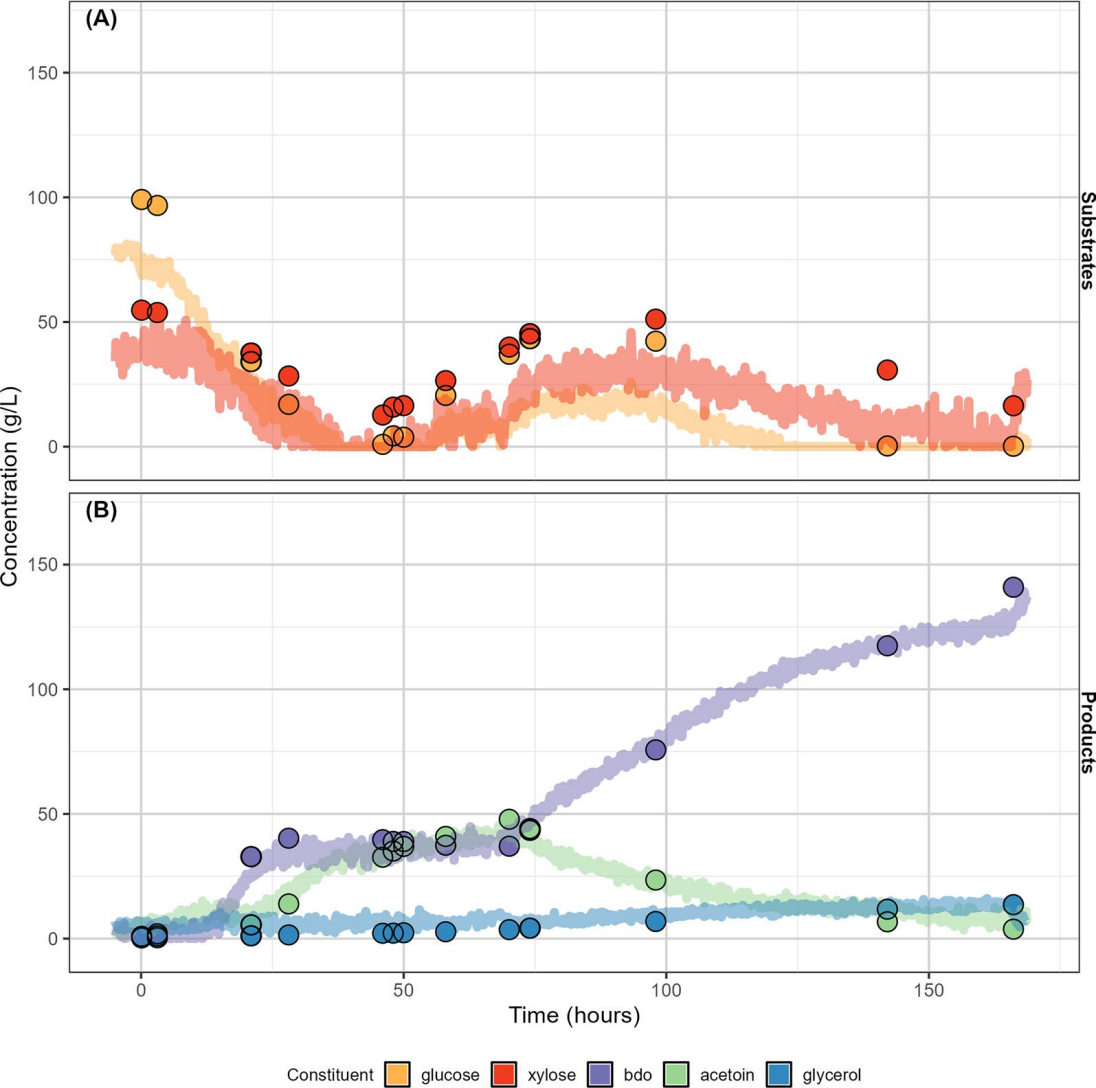
Performance of on-line calibration models—fermentation time course of a 10L pure sugar fermentation. Measured (points) and predicted (line) concentrations (g/L) of fermentation substrates (**A**) and products (**B**) for a unique 10 L fermentation experiment; neither model was trained on any spectra associated with this bioreactor volume. Despite the substantial differences in bioreactor geometry, the models show very good performance. To accommodate the larger volume bioreactor, the probe shaft dimensions were modified to fit the height of the larger vessel (405 mm length × 12 mm OD vs. 100 mm × 6.6 mm OD). The transflectance tip/fiber optic specifications were constant (6.4 mm OD tip with 2 mm transflectance pathlength with 2 × 600 µm fibers). To accommodate the larger volume bioreactor, the fiber length was increased from 2 m to 2.5 m

To evaluate the feasibility of using the on-line model in larger scale vessels, we applied the on-line model to a 10 L fermentation. Figure 7 shows the comparison of the on-line NIRS predictions of the substrates and products of a unique *Z. mobilis* fermentation fed using pure glucose and xylose to off-line HPLC measurements. During this fermentation, substrate feeding was inadvertently interrupted 34 h after inoculation, halting 2,3-BDO production and causing a plateau in measured 2,3-BDO concentration. During the loss of sugar feed, the glucose was fully consumed leaving xylose as the primary carbon source. When xylose is the primary sugar being consumed, the optimal DO concentration must be decreased to favor 2,3-BDO production over acetoin. Normally, this is done by reducing agitation, but this change in agitation did not occur due to the unplanned failure of the feed pump. The on-line NIRS prediction showed an increase in acetoin production during this time. Once the feed pump was restarted at approximately 46 h, it took an additional 24 h to re-establish conditions where glucose and xylose were not continually increasing and 2,3-BDO was the dominant product being produced. The on-line prediction model was able to accurately capture these events and inform decisions on sugar feed rates and agitation speeds to maximize 2,3-BDO production after the unexpected process upset, demonstrating the utility of on-line fermentation monitoring using NIRS.

The average prediction error (as evaluated by the comparison of RMSEP_unq_ in Supplemental Table S5) was larger for the experiment in the 10L reactor (Fig. 7) than the 500 mL reactor (Fig. 6), particularly for the sugar substrate concentrations. We believe this was due largely to the use of two different NIR probes, along with differences in the placement of the probes in the reactor. The best practice for model development is always to incorporate examples of the relationships one expects to observe within the calibration set; adding more 10 L experiments to the model calibration set would help determine the impacts of different probe geometries on model performance for 10 L fermentations.

## Conclusion

We have developed partial least squares (PLS) calibration models that successfully monitored 2,3-BDO fermentations with *Zymomonas mobilis* by measuring the concentrations of glucose, xylose, 2,3-BDO, acetoin, and glycerol in an at-line configuration, which is a useful tool for optimizing fermentation conditions in near real time. We have demonstrated that 2,3-BDO fermentations can also be monitored at-line using low-cost spectrometers and on-line using laboratory-grade spectrometers.

In addition, we presented a methodology or approach that can be effectively used by other researchers to evaluate the feasibility of NIRS/chemometrics for fermentation monitoring. By starting with a simple set of prepared samples at relevant concentrations, one can quickly identify the key spectral signatures of the analytes and determine if the limits of analyte identification and measurement via NIRS/chemometrics will be sufficient for practical use.

Then, by building at-line models using samples collected for off-line analysis, the complexity of the sample matrix as fermentation optimization proceeds can be captured, and one can begin to understand the additional complexities associated with sample matrix effects and realistic correlations among substrate and product concentrations, resulting in models that better capture relevant variability—a difficult task for a spectroscopic method known for its lack of peak resolution. A functioning at-line model still requires some sample preparation but eliminates the need for conventional time-consuming off-line analysis.

Finally, lessons learned from at-line approaches can be transferred on-line through the use of fiber optic probes. Building on-line models enables real-time monitoring and control, dramatically improving the effectiveness of fermentation development by eliminating the major analytical bottleneck—off-line conventional analysis.

Further work would involve integrating predictions into control systems to enable autonomous fermentation optimization (for example, adjusting substrate feeding rates or agitation speed in 2,3-BDO fermentations) and further evaluation of alternative low-cost spectrometers for easily field-deployable prediction systems. In addition, it will likely be valuable to more thoroughly investigate the practical issues associated with scaling across reactor size. This work included only 1 10 L run. As bioprocesses are scaled from shaker flask to industrial settings, methods for spectral collection will need to be reoptimized to account for the unique challenges that exist at each geometry. The increased complexity of a dataset that spans reactor geometries and volumes could lead to the necessity of using more complex model algorithms to properly capture the relationship between spectra and chemical information.

## Supplementary Information

Additional file 1.

## Data Availability

All NIRS spectroscopy and primary analytical data are available at https://data.nrel.gov/submissions/285. Modeling codes are available at https://github.com/NREL/nirbdoms.

## Abbreviations

2,3-BDO: 2,3-butanediol
HPLC: High-performance liquid chromatography
LAP: Laboratory analytical procedure
NIRS: Near-infrared spectroscopy
PCA: Principal component analysis
PLS: Partial least squares
RMSE: Root mean squared error
SNV: Standard normal variate
SG: Savitzky–Golay
