# Supplementary material for: Rapid monitoring of fermentations: a feasibility study on biological 2,3-butanediol production
Source: Biotechnol Biofuels Bioprod. 2025 Jun 7;18:60. doi: 10.1186/s13068-025-02662-1 (PMC12145592; doi:10.1186/s13068-025-02662-1)

***Supplementary Material for ‘Rapid monitoring of fermentations – a feasibility study on biological 2,3-butanediol production’***

**Table S1:** Summary of compositional analysis data of the major components for the *Zymomonas mobilis conversion* experiments for all three datasets. Lab-grade at-line denotes the set of filtered samples with spectra collected at-line using an optical grade quartz ring cup fit with a 0.2mm pathlength gold transflectance plate using an Antaris II FT-NIR spectrometer (Thermo-Fisher). Low-cost at-line denotes set of filtered samples with spectra collected at-line using an optical grade quartz ring cup fit with a 0.2mm pathlength gold transflectance plate using a NIRONE 2.5 hand-held spectrometer (Spectral Engines). Lab-grade on-line denotes the set of unfiltered samples collected in-line using a 2x600 µm broadband 0.22NA fiber-optic probe with a 2mm pathlength transflectance tip connected to a Antaris II FT-NIR spectrometer (Thermo-Fisher).

|  |  | Instrument | | | |
| --- | --- | --- | --- | --- | --- |
| Constituent | parameter | Lab-grade at-line | Low-cost at-line | | Lab-grade on- line |
|  | n | 700 | 363 | 112 | |
| Glucose (g/L) | mean | 30.13 | 29.79 | 40.35 | |
|  | median | 20.63 | 19.64 | 24.48 | |
|  | sd | 31.50 | 33.11 | 39.64 | |
|  | IQR | 51.02 | 52.30 | 71.20 | |
|  | min | 0.00 | 0.00 | 0.00 | |
|  | max | 125.05 | 125.05 | 131.58 | |
| Xylose (g/L) | mean | 28.02 | 23.76 | 33.55 | |
|  | median | 31.86 | 18.44 | 36.67 | |
|  | sd | 18.04 | 18.32 | 16.86 | |
|  | IQR | 31.34 | 31.91 | 28.28 | |
|  | min | 0.07 | 0.30 | 0.61 | |
|  | max | 71.37 | 66.72 | 61.33 | |
| 2,3-BDO (g/L) | mean | 37.56 | 25.40 | 45.78 | |
|  | median | 29.24 | 19.07 | 36.64 | |
|  | sd | 34.47 | 26.24 | 42.43 | |
|  | IQR | 50.33 | 32.07 | 56.64 | |
|  | min | 0.00 | 0.88 | 0.81 | |
|  | max | 169.57 | 138.81 | 143.73 | |
| Acetoin (g/L) | mean | 5.13 | 5.33 | 9.26 | |
|  | median | 1.53 | 1.15 | 3.83 | |
|  | sd | 7.25 | 7.77 | 11.26 | |
|  | IQR | 7.77 | 8.95 | 12.48 | |
|  | min | 0.00 | 0.05 | 0.10 | |
|  | max | 41.01 | 41.01 | 47.86 | |
| Glycerol (g/L) | mean | 4.11 | 2.75 | 5.33 | |
|  | median | 2.43 | 2.08 | 4.68 | |
|  | sd | 4.85 | 2.70 | 4.92 | |
|  | IQR | 3.95 | 3.70 | 6.73 | |
|  | min | 0.00 | 0.11 | 0.19 | |
|  | max | 22.74 | 13.10 | 21.90 | |

**Figure S1:** Box plots summarizing compositional analysis data of the major components within the *Z. mobilis* conversion of sugar to 2,3-BDO for all three datasets. Laboratory-grade at-line denotes the set of filtered samples with spectra collected at-line using an optical grade quartz ring cup fit with a 0.2mm pathlength gold transflectance plate on a Thermo Antaris II FT-NIR Spectrometer. Low-cost at-line denotes set of filtered samples with spectra collected at-line using an optical grade quartz ring cup fit with a 0.2mm pathlength gold transflectance plate using a NIRONE 2.5 hand-held spectrometer (Spectral Engines). Laboratory-grade on-line denotes the set of unfiltered samples collected in-line using a 2x600 µm broadband 0.22NA fiber optic transflectance dip probe fit with a 2mm pathlength tip configured to a Thermo Antaris II FT-NIR Spectrometer.
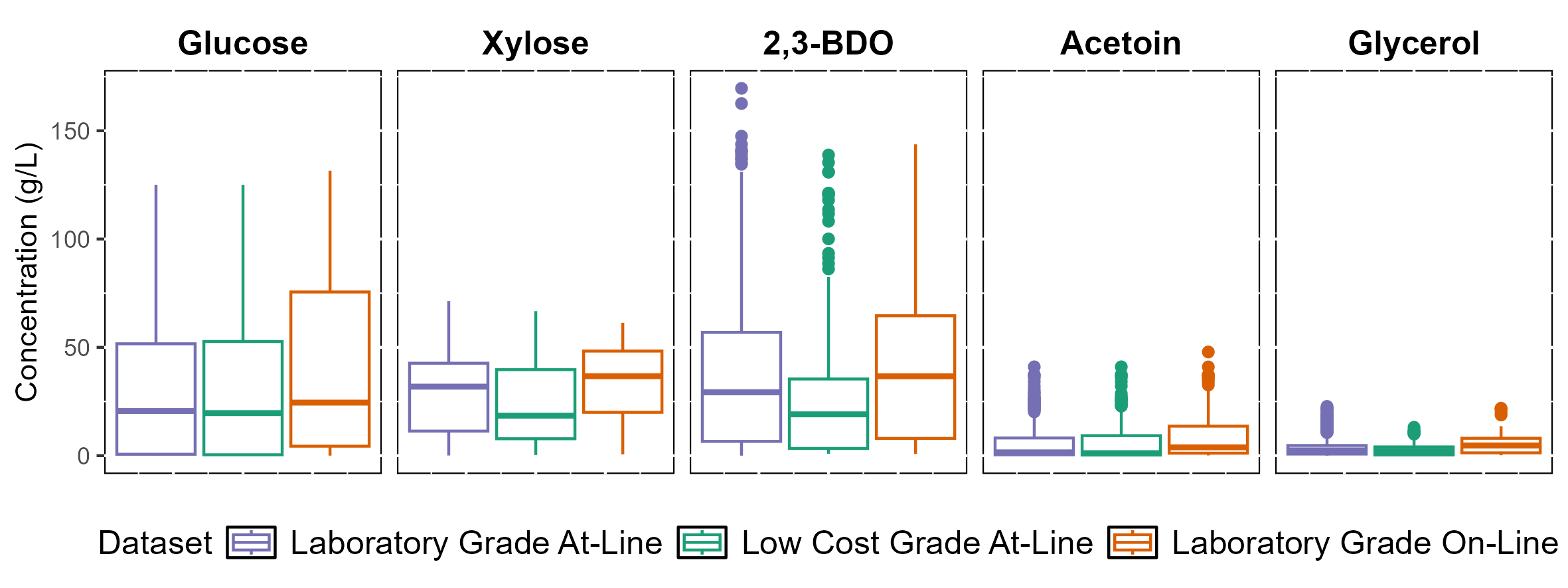


**Figure S2A:** Plots comparing correlations in constituent distributions in each of the three datasets used in this work


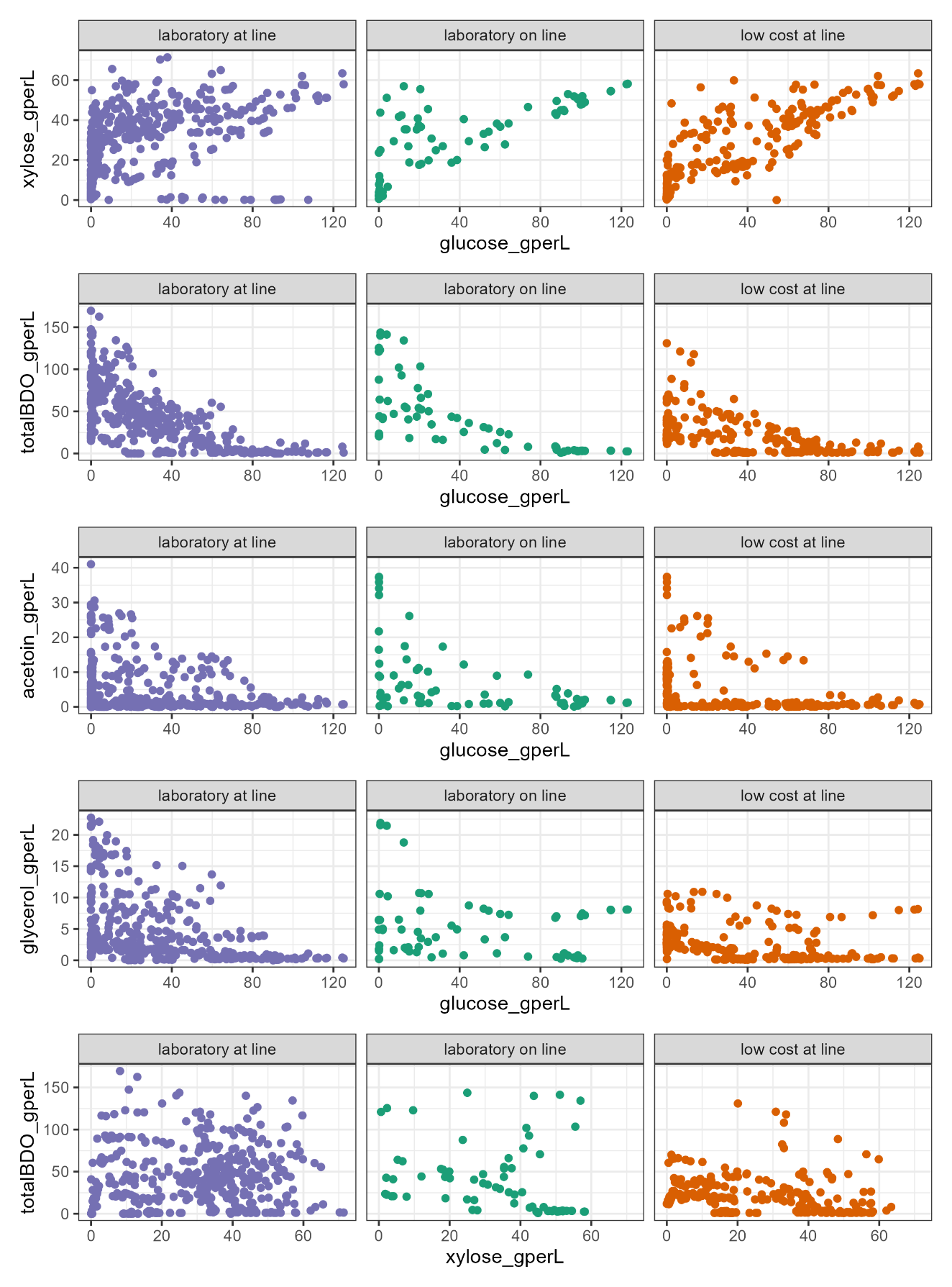


**Figure S2B:** Plots comparing correlations in constituent distributions in each of the three datasets used in this work


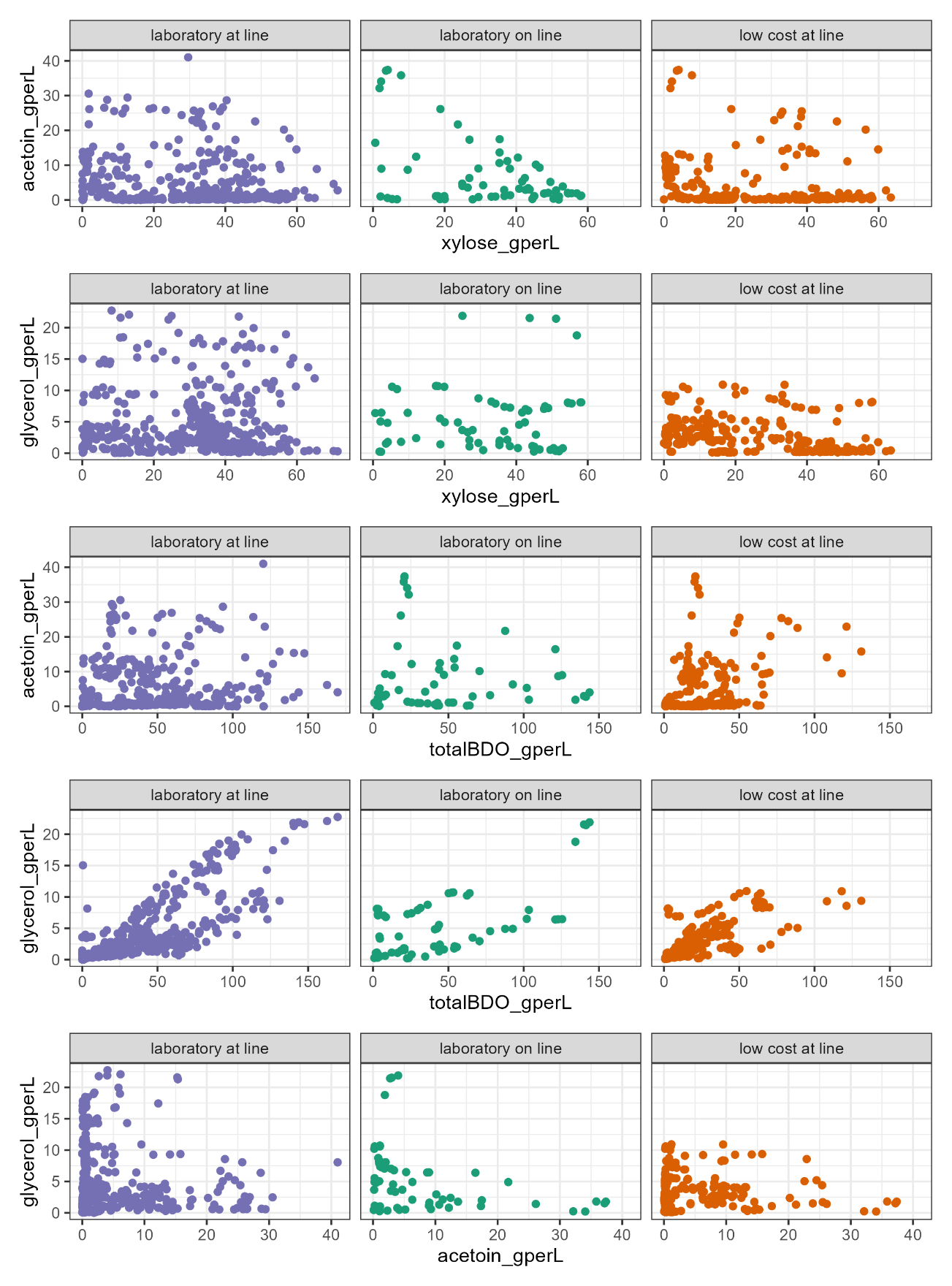


**Table S2 A:** Correlation coefficients (r2) among the chemical constituents in the *Z. mobilis* fermentation sample laboratory grade at-line NIR dataset

| **Parameter** | **Glucose** | **Xylose** | **Total BDO** | **Acetoin** | **Glycerol** |
| --- | --- | --- | --- | --- | --- |
| **Glucose** | 1.000 | 0.522 | -0.571 | -0.344 | -0.361 |
| **Xylose** |  | 1.000 | 0.019 | -0.216 | 0.028 |
| **Total BDO** |  |  | 1.000 | 0.144 | 0.776 |
| **Acetoin** |  |  |  | 1.000 | -0.105 |
| **Glycerol** |  |  |  |  | 1.000 |

**Table S2 B:** Correlation coefficients (r) among the chemical constituents in the *Z. mobilis* fermentation sample low cost grade at-line NIR dataset

| **Parameter** | **Glucose** | **Xylose** | **Total BDO** | **Acetoin** | **Glycerol** |
| --- | --- | --- | --- | --- | --- |
| **Glucose** | 1.000 | 0.694 | -0.696 | -0.474 | -0.186 |
| **Xylose** |  | 1.000 | -0.200 | -0.403 | 0.021 |
| **Total BDO** |  |  | 1.000 | 0.071 | 0.633 |
| **Acetoin** |  |  |  | 1.000 | -0.315 |
| **Glycerol** |  |  |  |  | 1.000 |

**Table S2 C:** Correlation coefficients (r) among the chemical constituents in the *Z. mobilis* fermentation sample laboratory grade on-line NIR dataset

| **Parameter** | **Glucose** | **Xylose** | **Total BDO** | **Acetoin** | **Glycerol** |
| --- | --- | --- | --- | --- | --- |
| **Glucose** | 1.000 | 0.808 | -0.463 | -0.381 | -0.340 |
| **Xylose** |  | 1.000 | -0.061 | -0.155 | -0.239 |
| **Total BDO** |  |  | 1.000 | 0.500 | 0.741 |
| **Acetoin** |  |  |  | 1.000 | 0.191 |
| **Glycerol** |  |  |  |  | 1.000 |

**Table S3** Summary of model performance results for at-line modeling. All at-line modeling spectra were obtained using filtered fermentation liquors scanned in optical grade quartz ring cups fit with a 0.2mm pathlength gold transflectance head. Three different models are compared- Laboratory-grade at-line FS - model built from spectra collected using a Thermo Antaris II FT-NIR Spectrometer with the fully usable signal range from 4000 to 6100 cm-1. Laboratory-grade at-line Truncated low-cost feasibility- model built from the Laboratory-grade at-line dataset with spectra but truncated to 4082-5000 cm -1 to match the spectral range of the Low-cost spectrometer. Low-cost at-line – model built from spectra collected using a handheld NIRONE 2.5 spectrometer (Spectral Engines). For all three models, one unique experiment was held out of the population. RMSEC - root mean squared error of calibration; RMSECV - root mean squared error of cross validation; RMSEP - root mean squared error of prediction for the independent validation set; RMSEPunq - root mean squared error of prediction for the unique experiment; R2 - coefficient of determination. To test the significance of differences observed between model performance parameters, we used a Fisher z-transform followed by a Studentized t-test to compare the difference between the transformed correlation coefficients (α=0.05). We used an F test to compare normalized RMSE values (α=0.05). (43) * denotes model performance parameter that is statistically significantly different from laboratory grade at-line model parameter of the same constituent. ˘ denotes model performance parameter that is statistically significantly different from the performance of BDO for the same model type. Due to the confounding nature of calibration range, RMSE without normalization was not tested for statistical significance.

|  |  | Glucose | | | Xylose | | | 2,3-BDO | | | | Acetoin | | | Glycerol | | |
| --- | --- | --- | --- | --- | --- | --- | --- | --- | --- | --- | --- | --- | --- | --- | --- | --- | --- |
|  | **Performance Parameter** | Laboratory Grade At-Line | Laboratory Grade At-Line Low cost feasibility | Low Cost at line | Laboratory Grade At-Line | Laboratory Grade At-Line Low cost feasibility | Low Cost at line | Laboratory Grade At-Line | Laboratory Grade At-Line Low cost feasibility | Low Cost at line | Laboratory Grade At-Line | | Laboratory Grade At-Line Low cost feasibility | Low Cost at line | Laboratory Grade At-Line | Laboratory Grade At-Line Low cost feasibility | Low Cost at line |
| As-calculated  (g/L) | RMSEC | 4.12 | 4.97 | 8.29 | 2.66 | 3.05 | 5.59 | 3.21 | 3.01 | 3.33 | 0.92 | | 1.15 | 3.18 | 0.90 | 1.26 | 1.26 |
|  | RMSECV | 4.47 | 5.17 | 8.93 | 2.88 | 3.28 | 6.17 | 3.33 | 3.15 | 3.95 | 0.97 | | 1.20 | 3.84 | 0.97 | 1.32 | 1.44 |
|  | RMSEP | 3.93 | 4.71 | 7.51 | 2.86 | 2.55 | 6.41 | 2.28 | 1.62 | 3.96 | 0.79 | | 1.02 | 3.73 | 0.73 | 1.18 | 1.21 |
|  | RMSEP_UNQ_ | 2.89 | 4.27 | 7.93 | 2.25 | 2.26 | 9.28 | 3.21 | 2.42 | 3.06 | 0.65 | | 0.57 | 2.96 | 0.67 | 0.74 | 2.14 |
| Normalized to calibration set concentration range (unitless) | RMSEC | 0.03 | 0.04 | 0.07 | 0.04 | 0.04 | 0.09 | 0.02 | 0.02 | 0.03 | 0.02 | | 0.03˘ | 0.09 | 0.04 | 0.06 | 0.12 |
|  | RMSECV | 0.04 | 0.04 | 0.07 | 0.04 | 0.05 | 0.10 | 0.02 | 0.02 | 0.03 | 0.02 | | 0.03 | 0.10 | 0.04 | 0.06 | 0.13 |
|  | RMSEP | 0.03 | 0.04 | 0.06 | 0.04 | 0.04* | 0.10 | 0.01 | 0.01* | 0.03 | 0.02 | | 0.02 | 0.10 | 0.03 | 0.05 | 0.11 |
|  | RMSEP_UNQ_ | 0.02 | 0.03 | 0.06 | 0.03 | 0.03 | 0.15 | 0.02 | 0.01* | 0.02 | 0.02 | | 0.01* | 0.08 | 0.03 | 0.03 | 0.20 |
| As-calculated | R^2^C | 0.98 | 0.98* | 0.94* | 0.98 | 0.96 | 0.91* | 0.99 | 0.99 | 0.98* | 0.98 | | 0.97* | 0.82* | 0.97 | 0.95* | 0.77* |
|  | R^2^CV | 0.98 | 0.97* | 0.93* | 0.98 | 0.96 | 0.89* | 0.99 | 0.99 | 0.97* | 0.98 | | 0.97* | 0.72* | 0.96 | 0.94* | 0.70* |
|  | R^2^P | 0.99 | 0.98* | 0.94* | 0.98 | 0.97 | 0.89* | 1.00 | 1.00* | 0.98 | 0.98 | | 0.98* | 0.86* | 0.97 | 0.90 | 0.70* |
|  | R^2^P_UNQ_ | 0.99 | 0.99* | 0.96* | 0.99 | 0.99 | 0.76* | 1.00 | 1.00 | 1.00* | 0.98 | | 0.99 | 0.75* | 0.99 | 0.98* | 0.98* |

**Table S4.** Summary of model performance results comparing the at-line to on-line spectral data collection configurations using the same spectral range. Both datasets were collected using a Thermo Antaris II FT-NIR Spectrometer. Laboratory-grade at-line PS represents the at-line model obtained using filtered fermentation liquors scanned in optical grade quartz ring cups fit with a 0.2mm pathlength gold transflectance. Laboratory-grade on-line represents the on-line model obtained from spectra using a 2x600 µm broadband 0.22NA fiber-optic probe fit with a 2mm transflectance pathlength tip. In both models, the spectral regions from 5348-6578 cm^-1^ and 4377 to 4700 cm^-1^ were used. In both datasets, one unique experiment was held out of the population validation. RMSEC - root mean squared error of calibration; RMSECV - root mean squared error of cross validation; RMSEP - root mean squared error of prediction for the independent validation set; RMSEP_unq_ - root mean squared error of prediction for the unique experiment; R^2^ - coefficient of determination. To test the significance of differences observed between model performance parameters, we used a Fisher z-transform followed by a Studentized t-test to compare the difference between the transformed correlation coefficients (α=0.05). We used an F test to compare normalized RMSE values (α=0.05). (43) * denotes model performance parameter that is statistically significantly different from laboratory grade at-line on-line feasibility model parameter of the same constituent ˘ denotes model performance parameter that is statistically significantly different from the performance of BDO for the same model type. Due to the confounding nature of calibration range, RMSE without normalization was not tested for statistical significance.

|  |  | Glucose | | Xylose | | 2,3-BDO | | Acetoin | | Glycerol | |
| --- | --- | --- | --- | --- | --- | --- | --- | --- | --- | --- | --- |
| As-calculated  (g/L) | Performance Parameter | Laboratory Grade At-Line + On-Line Feasibility | Laboratory Grade On-Line | Laboratory Grade At-Line + On-Line Feasibility | Laboratory Grade On-Line | Laboratory Grade At-Line + On-Line Feasibility | Laboratory Grade On-Line | Laboratory Grade At-Line + On-Line Feasibility | Laboratory Grade On-Line | Laboratory Grade At-Line + On-Line Feasibility | Laboratory Grade On-Line |
|  | RMSEC (g/L) | 3.74 | 7.60 | 3.06 | 3.16 | 2.05 | 2.66 | 1.12 | 1.38 | 0.92 | 1.22 |
|  | RMSECV(g/L) | 3.98 | 9.11 | 3.15 | 5.52 | 2.11 | 3.74 | 1.16 | 2.06 | 0.97 | 1.90 |
|  | RMSEP(g/L) | 3.75 | 5.57 | 2.79 | 5.09 | 1.59 | 3.20 | 1.09 | 1.65 | 0.93 | 1.01 |
|  | RMSEP_UNQ_ (g/L) | 2.03 | 6.71 | 1.56 | 11.29 | 2.14 | 5.56 | 0.62 | 3.52 | 0.65 | 2.88 |
| Normalized to calibration set concentration range (unitless) | RMSEC (g/L) | 0.03 | 0.06* | 0.04 | 0.05 | 0.01 | 0.02* | 0.03 | 0.04 | 0.04 | 0.06 |
|  | RMSECV(g/L) | 0.03 | 0.07* | 0.04 | 0.10 | 0.01 | 0.03* | 0.03 | 0.06 | 0.04 | 0.09 |
|  | RMSEP(g/L) | 0.03 | 0.05 | 0.04 | 0.09 | 0.01 | 0.02* | 0.03 | 0.04 | 0.04 | 0.05 |
|  | RMSEP_UNQ_ (g/L) | 0.02 | 0.05* | 0.02 | 0.20* | 0.01 | 0.04* | 0.02 | 0.09 | 0.03 | 0.13 |
| As calculated | R^2^C | 0.98 ˘ | 0.97* ˘ | 0.97 ˘ | 0.97 ˘ | 1.00* | 1.00* | 0.98* ˘ | 0.98 ˘ | 0.97 ˘ | 0.94* ˘ |
|  | R^2^CV | 0.98 ˘ | 0.95* ˘ | 0.97 ˘ | 0.90* ˘ | 1.00* | 0.99 | 0.97* ˘ | 0.96* ˘ | 0.97 ˘ | 0.86* ˘ |
|  | R^2^P | 0.99 ˘ | 0.99 ˘ | 0.98 ˘ | 0.94* ˘ | 1.00* | 0.99 | 0.98* ˘ | 0.98 ˘ | 0.94* ˘ | 0.96 ˘ |
|  | R^2^P_UNQ_ | 1.00* ˘ | 0.97* ˘ | 0.98* ˘ | 0.75* ˘ | 1.00 | 1.00 | 0.99* ˘ | 0.81* ˘ | 0.99* ˘ | 0.95* ˘ |

**Figure S3**: Predicted vs Measured Plots for RMSECV (column 1), RMSEP (column 2), and RMSEP_unq_ (column 3) validation sets evaluating at-line model performance for Glucose prediction. Three at-line models are shown – laboratory grade at-line full spectra Model (row 1), laboratory grade at-line low-cost feasibility test range Model (row 2), and low-cost at-line Model (row 3). Measurements are colored by sugar source.
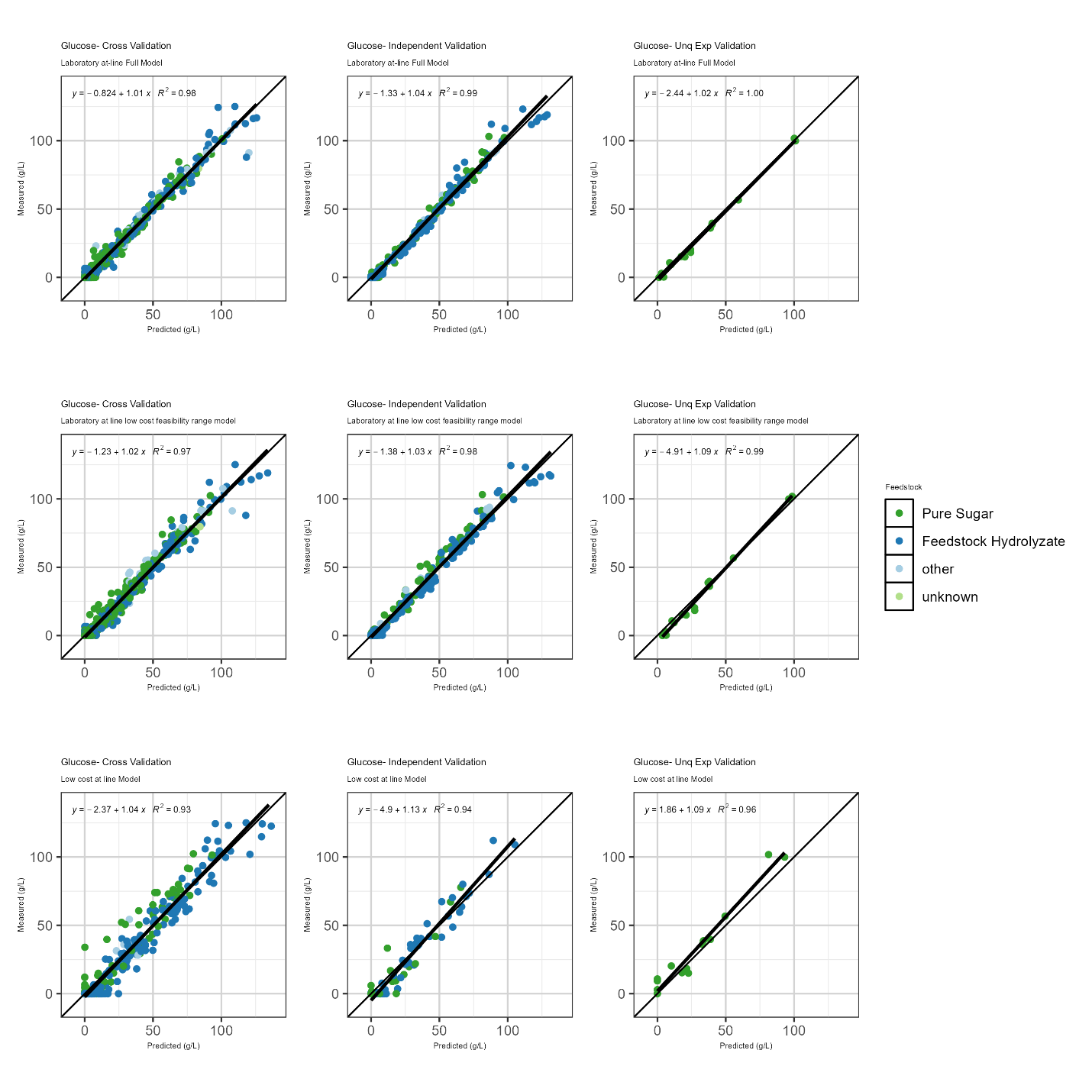


**Figure S4:** Predicted vs Measured Plots for RMSECV (column 1), RMSEP (column 2), and RMSEP_unq_ (column 3) validation sets evaluating at-line model performance for Xylose prediction. Three at-line models are shown – laboratory grade at-line full spectra Model (row 1), laboratory grade at-line low-cost feasibility test range Model (row 2), and low-cost at-line Model (row 3). Measurements are colored by sugar source.
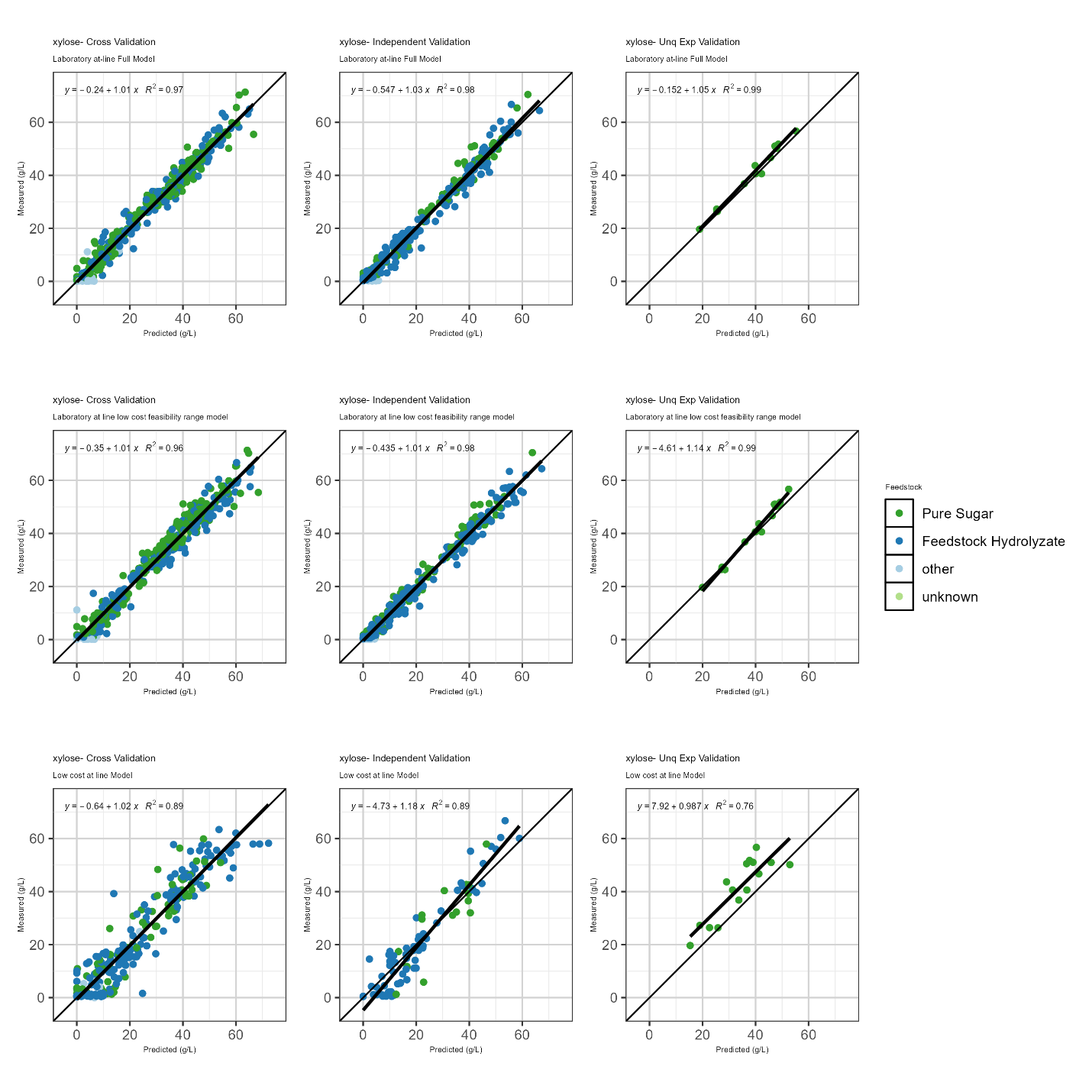


**Figure S5:** Predicted vs Measured Plots for RMSECV (column 1), RMSEP (column 2), and RMSEP_unq_ (column 3) validation sets evaluating at-line model performance for total BDO prediction. Three at-line models are shown – laboratory grade at-line full spectra Model (row 1), laboratory grade at-line low-cost feasibility test range Model (row 2), and low-cost at-line Model (row 3). Measurements are colored by sugar source.
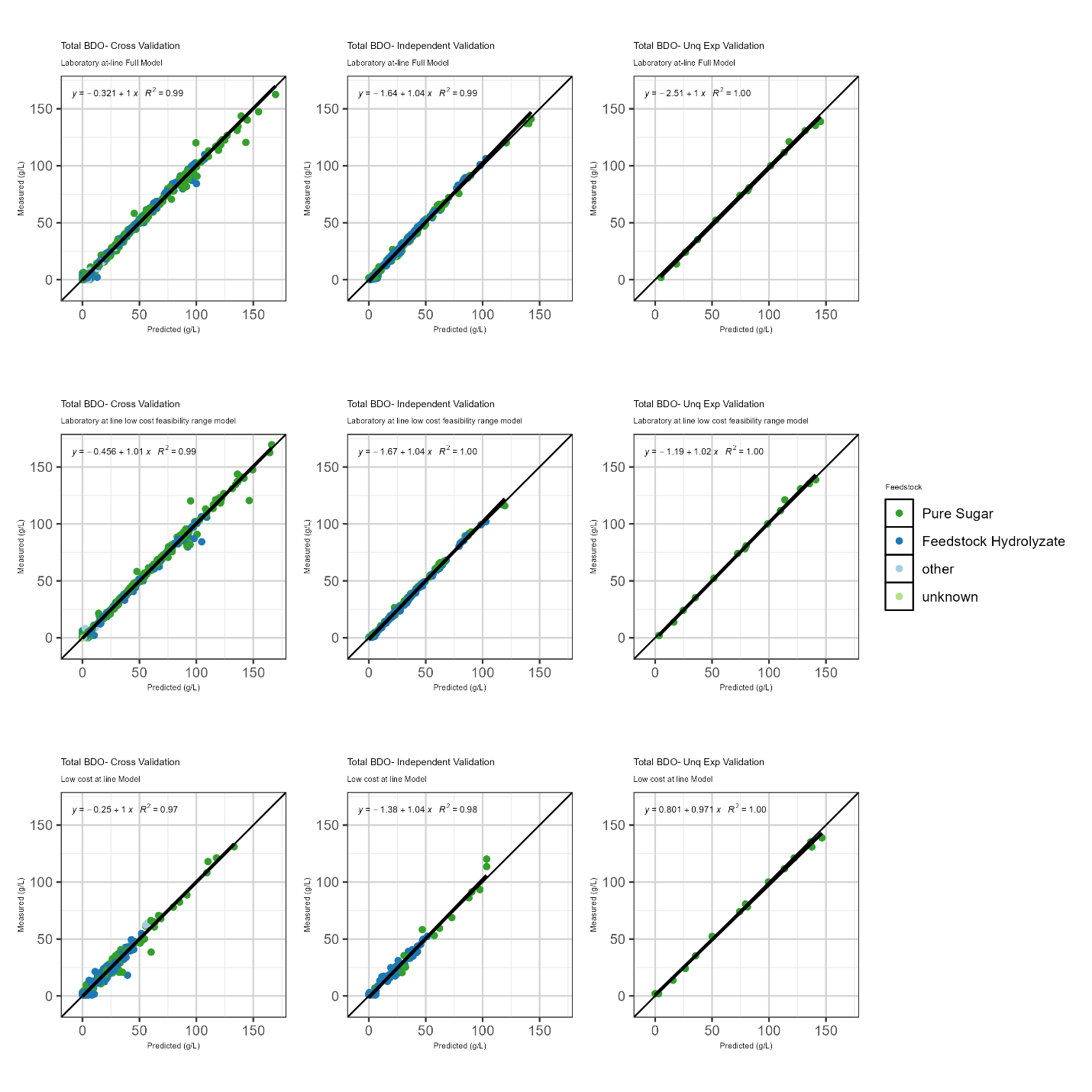


**Figure S6:** Predicted vs Measured Plots for RMSECV (column 1), RMSEP (column 2), and RMSEP_unq_ (column 3) validation sets evaluating at-line model performance for total acetoin prediction. Three at-line models are shown – laboratory grade at-line full spectra Model (row 1), laboratory grade at-line low-cost feasibility test range Model (row 2), and low-cost at-line Model (row 3). Measurements are colored by sugar source.
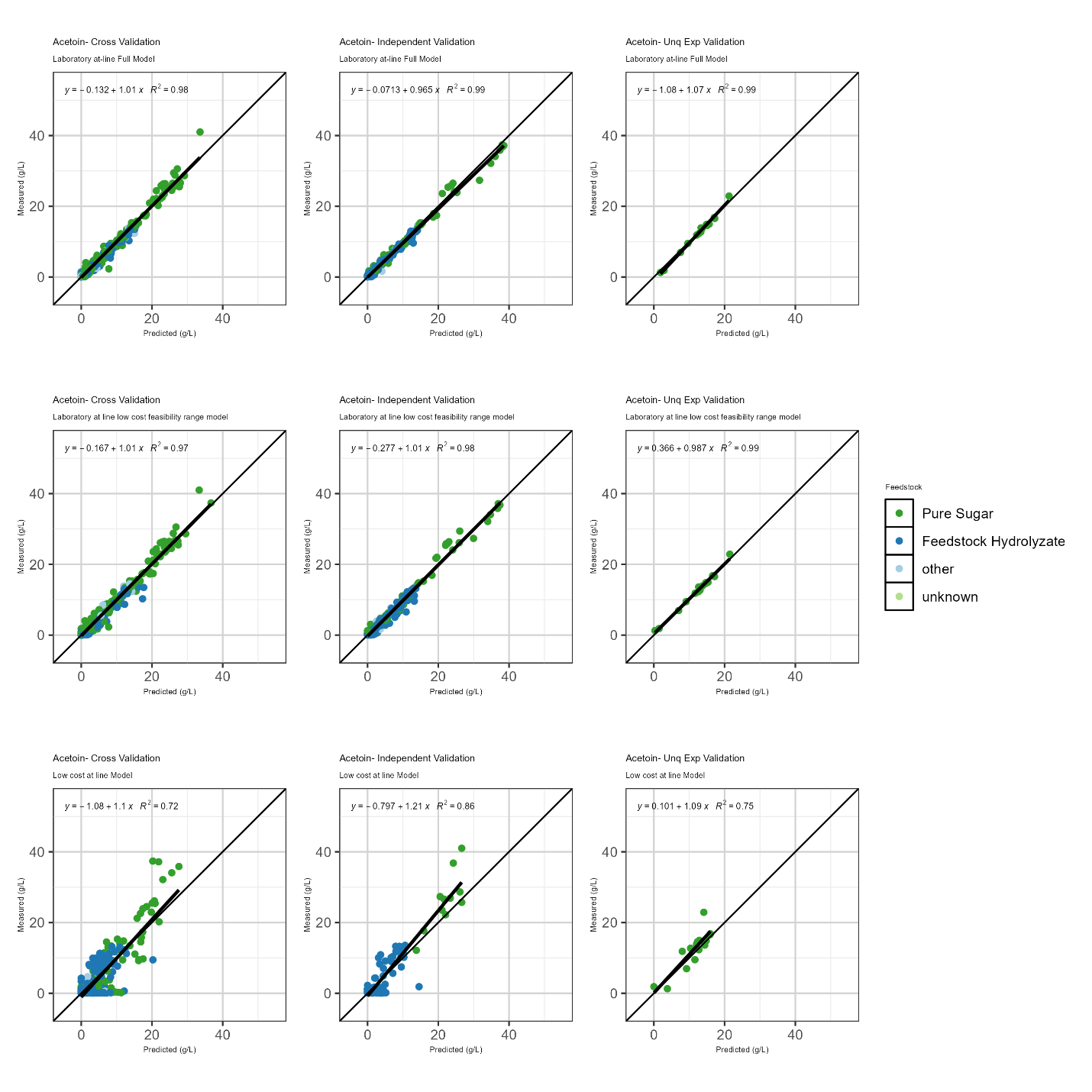


**Figure S7:** Predicted vs Measured Plots for RMSECV (column 1), RMSEP (column 2), and RMSEP_unq_ (column 3) validation sets evaluating at-line model performance for glycerol prediction. Three at-line models are shown – laboratory grade at-line full spectra Model (row 1), laboratory grade at-line low-cost feasibility test range Model (row 2), and low-cost at-line Model (row 3). Measurements are colored by sugar source.
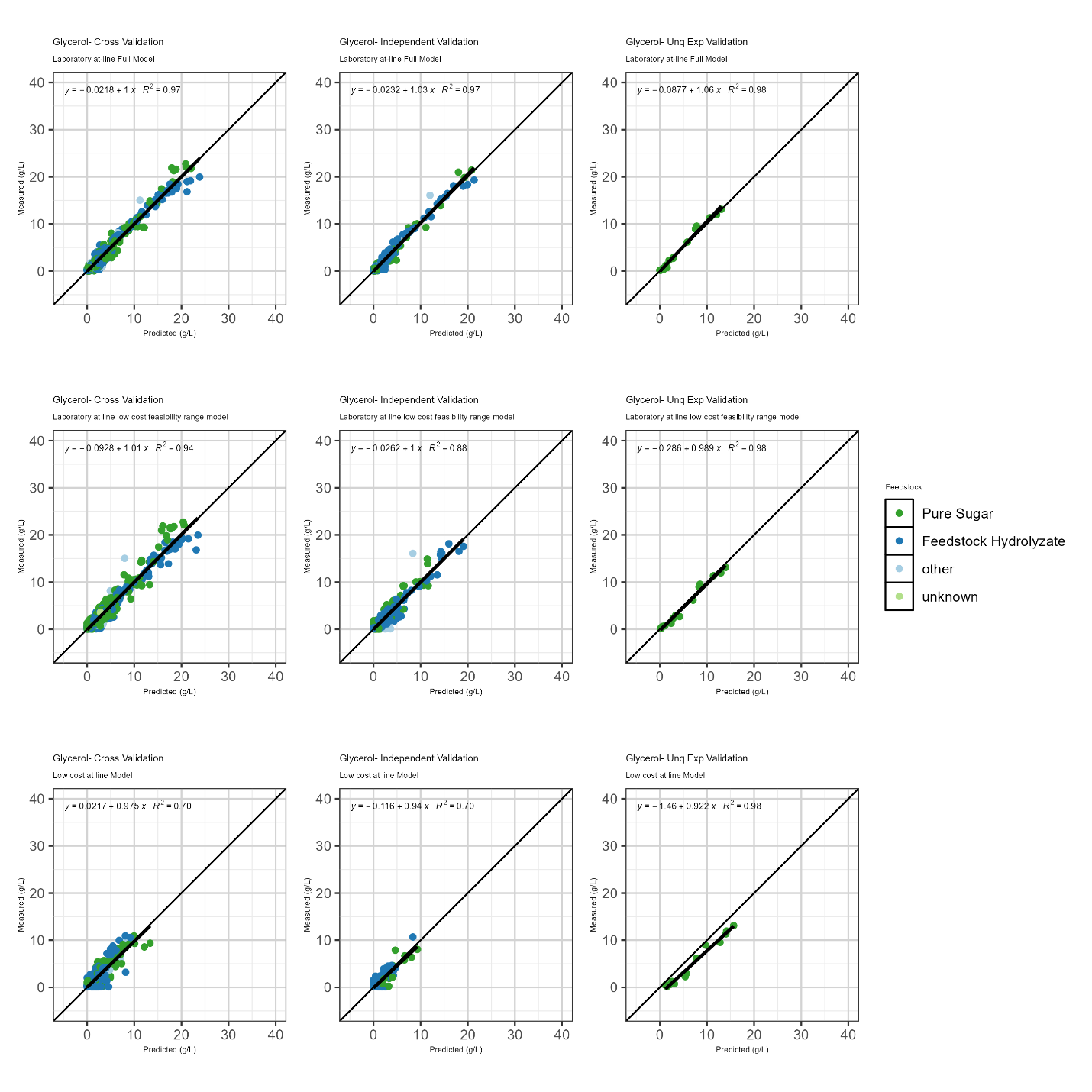


**Figure S8:** Predicted vs Measured Plots for RMSECV (column 1), RMSEP (column 2), and RMSEP_unq_ (column 3) validation sets evaluating at-line model performance using the laboratory-grade at-line dataset with spectra truncated to the laboratory-grade on-line optimized spectral range. Each row evaluates a different constituent (glucose (row 1), xylose (row 2), total BDO (row 3), acetoin (row 4), and glycerol (row 5)). Measurements are colored by sugar source.


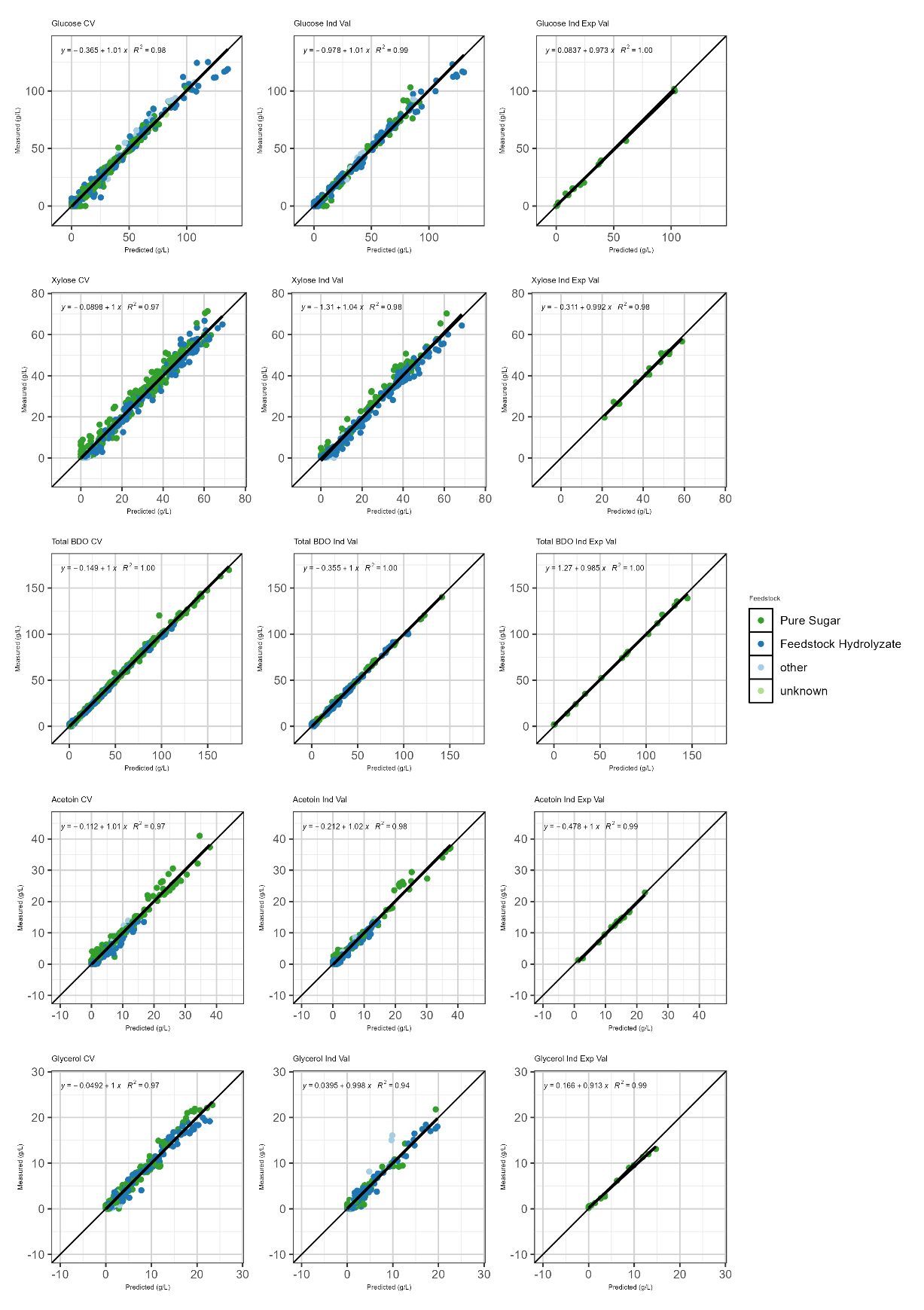


**Figure S9:** Predicted vs Measured Plots for RMSECV (column 1), RMSEP (column 2), and RMSEP_unq_ (column 3) validation sets evaluating laboratory-grade in-line model performance using the Thermo B dataset. Each row evaluates a different constituent (glucose (row 1), xylose (row 2), total BDO (row 3), acetoin (row 4), and glycerol (row 5)). Measurements are colored by sugar source.


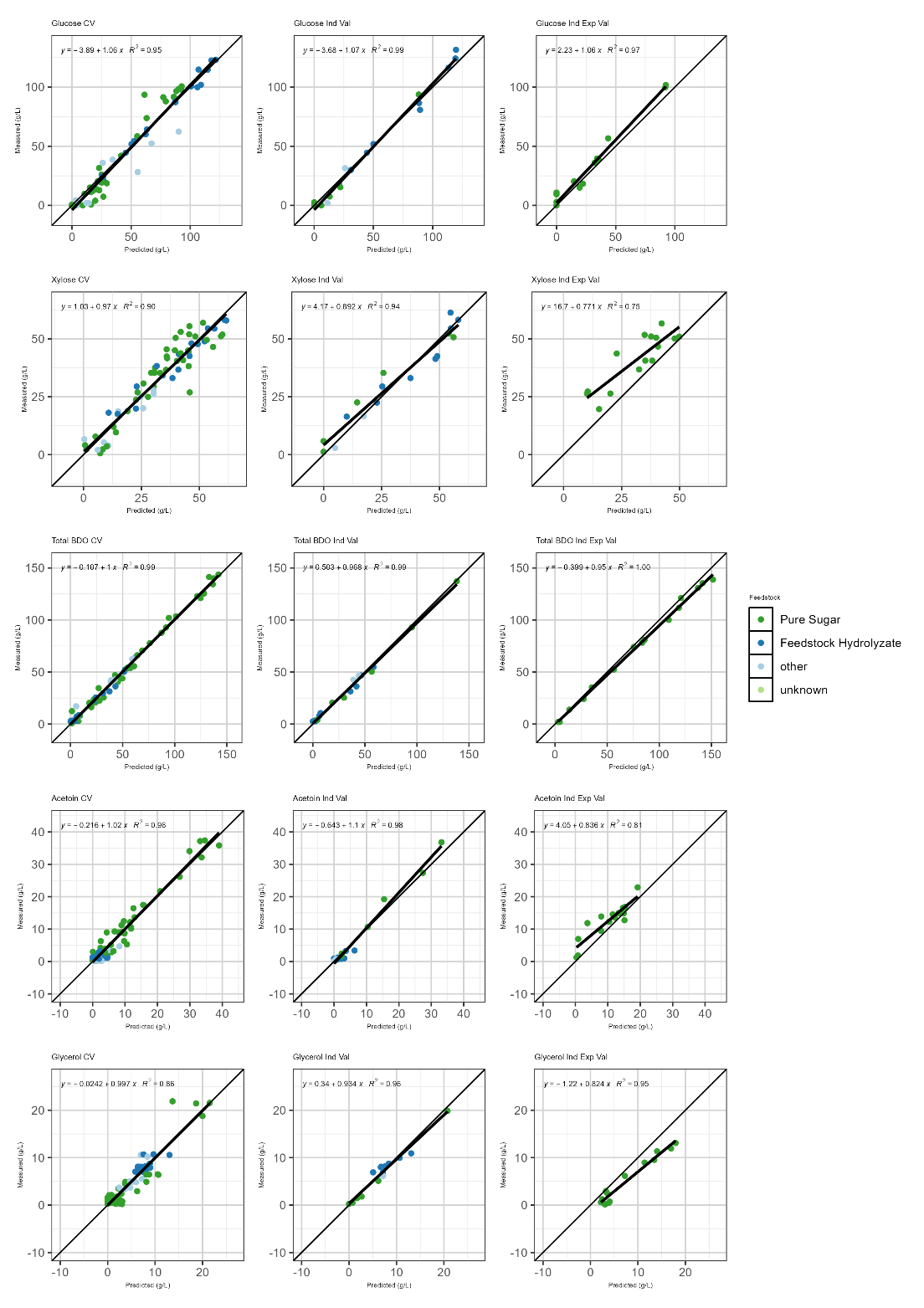


**Figure S10**: Plots of predictions over time of fermentation for 9 unique fermentations that make up the laboratory-grade on-line calibration dataset. Each unique experiment is split up into two frames, where 1 denotes the measurements for the fermentation reactants, glucose and xylose, while 2 denotes the measurements for the fermentation products, BDO, acetoin, and glycerol. The darker points represent the HPLC measured concentrations of each sample at a given timepoint. The lighter lines represent the NIR predictions.
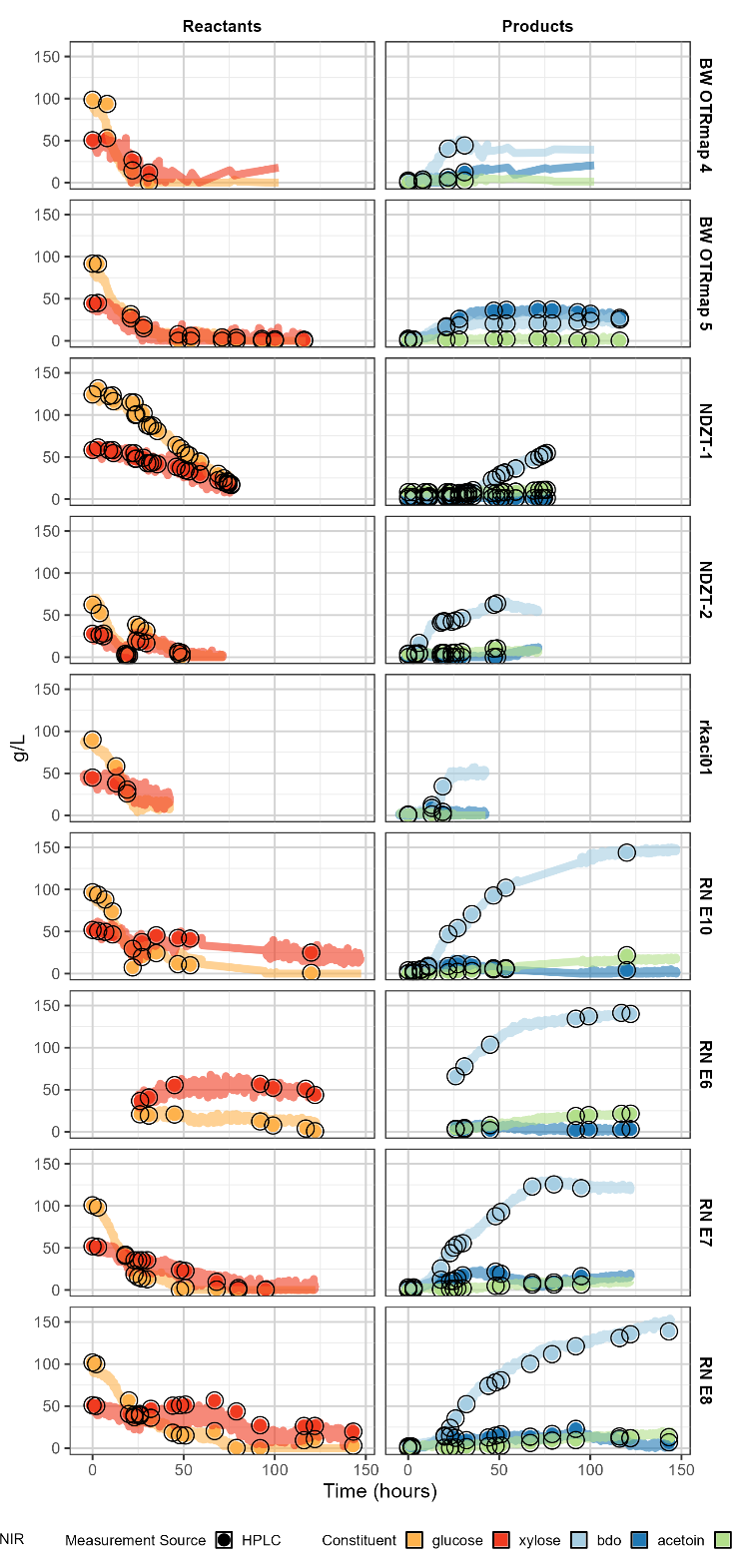


**Table S5: Comparing RMSEP_UNQ_ performance for on-line experiment**

Table comparing the calculated RMSE_unq_ values for at-line and on-line models, starting with the least complex system (predicting at line samples using the laboratory grade at-line modle) to the most complext system (predicting on line samples inside a 10L fermenter using the laboratory grade on-line model built for 0.5L fermentations). Each RMSE is calculated from samples collected across the timespan of a unique fermentation (from inoculation to end of data collection). No timepoint samples from the unique fermentation are included in the calibration data for building models.

|  |  | Constituent | | | | |
| --- | --- | --- | --- | --- | --- | --- |
| **Model** | **Unique validation dataset domain description** | **Glucose** | **Xylose** | **2,3-BDO** | **Acetoin** | **Glycerol** |
| Laboratory Grade At-Line | Laboratory Grade At-Line | **2.89** | **2.25** | **3.21** | **0.65** | **0.67** |
| Laboratory Grade At-Line On-Line Feasibility | Laboratory Grade At-Line On-Line Feasibility | **2.03** | **1.56** | **2.14** | **0.62** | **0.65** |
| Laboratory Grade On-Line 1.5L | Laboratory Grade On-Line 1.5L | **6.71** | **11.29** | **5.56** | **3.52** | **2.88** |
|  | Laboratory Grade On-Line 10L | **34.86** | **16.01** | **4.55** | **4.48** | **3.85** |

**Figure S11: Results of Spectral Noise Analysis:**

Top figure shows the standard material spectral signatures in the combinational region, as previously shown and described in figure 2. Transformed transflectance spectra (zoomed in to regions of high signal) for aqueous standards of150g/L glucose, 150g/L xylose, 150g/L 2,3-BDO, 100g/L acetoin, and 100g/L glycerol collected using the laboratory-grade at-line data collection method are shown. The dashed grey vertical lines represent the selected wavelengths used for spectral noise comparison between the two instruments which correspond with high spectral activity among the standard materials, indicating their potential importance to modeling. The bottom figure shows the differences in standard deviation of the signal response in the low cost instrument (blue) vs the laboratory grade instrument (red) across water control spectra (n = 20) taken across the scanning campaign using the at-line configuration. The Low-cost spectra have higher standard deviation across the entire combinational region. To account for differences in spectral resolution between the instruments, the standard deviations across the 10 wavenumbers highlighted by the vertical dashed grey lines were averaged. The result provided a relative noise comparison between the two instruments. The text on the plot shows illustrates the difference in noise between the two instruments as a result of this calculation.


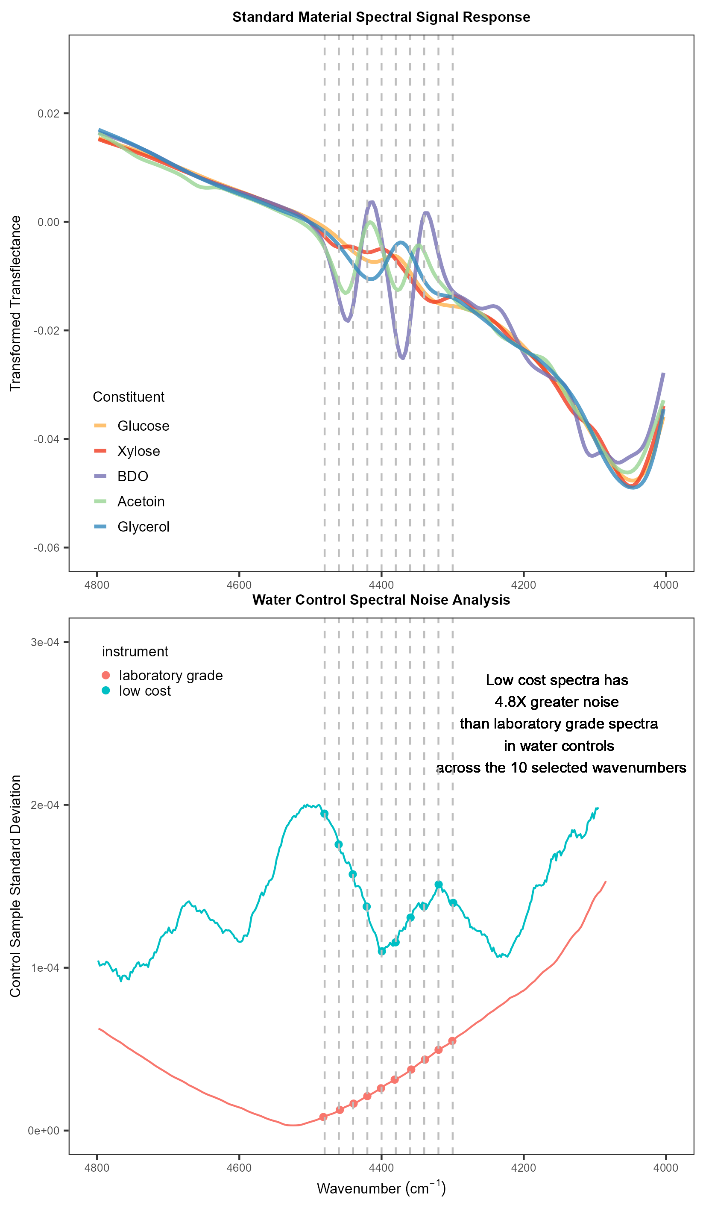

Supplement: Supplementary file 1 — Additional file 1. [file 13068_2025_2662_MOESM1_ESM.docx]
